# Synthesis of Black Phosphorene Quantum Dots from Red Phosphorus**

**DOI:** 10.1002/chem.202301232

**Published:** 2023-09-07

**Authors:** Rebecca R. C. Shutt, Thrinathreddy Ramireddy, Evgenios Stylianidis, Camilla Di Mino, Rebecca A. Ingle, Gabriel Ing, Ary A. Wibowo, Hieu T. Nguyen, Christopher A. Howard, Alexey M. Glushenkov, Andrew Stewart, Adam J. Clancy

**Affiliations:** ^1^ Department of Physics and Astronomy University College London London WC1E 6BT UK; ^2^ Research School of Chemistry The Australian National University Acton ACT 2601 Australia; ^3^ Battery Storage and Grid Integration Program The Australian National University Acton ACT 2601 Australia; ^4^ Department of Chemistry University College London London WC1E 6BT UK; ^5^ School of Engineering The Australian National University Acton ACT 2601 Australia

**Keywords:** BPQDs, liquid-phase TEM, LP-TEM, phosphorene quantum dots, PQDs

## Abstract

Black phosphorene quantum dots (BPQDs) are most commonly derived from high‐cost black phosphorus, while previous syntheses from the low‐cost red phosphorus (P_red_) allotrope are highly oxidised. Herein, we present an intrinsically scalable method to produce high quality BPQDs, by first ball‐milling P_red_ to create nanocrystalline P_black_ and subsequent reductive etching using lithium electride solvated in liquid ammonia. The resultant ~25 nm BPQDs are crystalline with low oxygen content, and spontaneously soluble as individualized monolayers in tertiary amide solvents, as directly imaged by liquid‐phase transmission electron microscopy. This new method presents a scalable route to producing quantities of high quality BPQDs for academic and industrial applications.

## Graphical abstract

**Black phosphorene quantum dots** can now be made at remarkably low cost in a simple two step process. Ball milling cheap abundant red phosphorus creates quantu dots trapped in bulk, which can then be freed by etching the surrounding amorphous phosphorus with lithium electride solution.

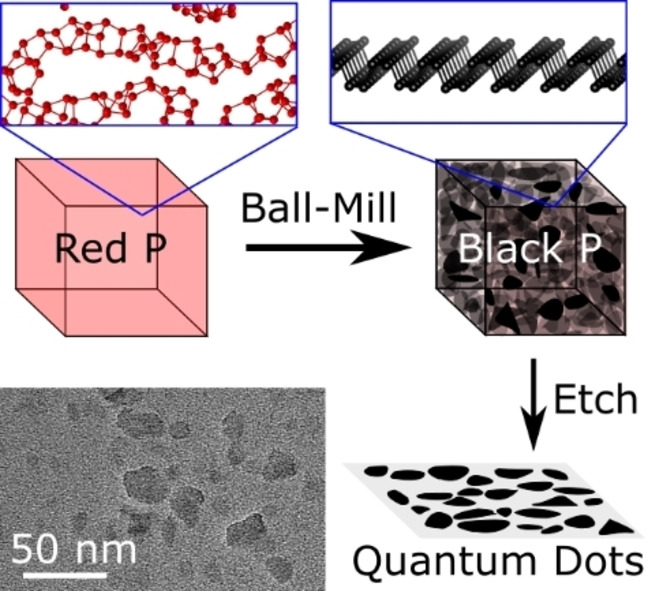

## Introduction

Black phosphorous (P_black_) is an allotrope of phosphorous and a van der Waals layered material, consisting of corrugated layers of phosphorous where each P atom is connected to three surrounding P atoms via two short P−P bonds and one long P−P bond. Nanomaterials isolated from the parent 3D P_black_, such as phosphorene (2D), phosphorene nanoribbons (PNRs, 1D), and black phosphorene quantum dots (BPQDs, 0D) have shown promising applications in energy harvesting and storage.^[1]^

While “bottom‐up” syntheses are under development,^[2]^ 2D phosphorene is most commonly synthesized through “top‐down” exfoliation of its 3D counterpart, P_black_, through either the so‐called scotch‐tape method, or liquid phase exfoliation typically through sonication in a suitable solvent/surfactant solution. For the latter route, the sonication provides sufficient shear force to break the interlayer van der Waals interactions to free individual 2D sheets, with reagglomeration kinetically hindered by adsorbed solvent/surfactant.

More aggressive sonication of P_black_ has led to the production of BPQDs,^[3]^ a 0D material with <100 nm lateral sheet sizes with the resultant in‐plane quantum confinement modulating the properties. The practical properties of BPQDs may be tuned through functionalization reactions,^[4]^ edge chemistry,^[5]^ and dielectric environment,^[6]^ and they have shown promise in a range of application including saturatable absorbers,^[7]^ photovoltaics,^[5]^ batteries,^[8]^ biocompatible photothermal agents,^[9]^ lubricants,^[10]^ and ultrafast photonics.^[11]^ Owing to phosphorus’ rich array of allotropes, BPQDs are part of a wider family of phosphorus‐based quantum dots, providing a wide array of chemistries and properties suited to next generation applications including violet (aka Hittorfene) phosphorus quantum dots,^[12]^ fibrous phosphorus quantum dots,^[13]^ and even pseudo‐spherical red phosphorus quantum dots.^[14]^

Like all phosphorus allotropes, P_black_ is not found naturally. Pure phosphorus is produced via the ‘thermal process’, reducing phosphate minerals to form white phosphorus P_4_ molecules.^[15]^ Under heating (or under UV), P_4_ is converted to the non‐toxic, non‐pyrophoric red phosphorus (P_red_), which is amorphous, consisting of a multitude of crosslinked phosphorus chains and clusters.^[16]^ Thousands of tons of P_red_ are produced each year and it is available from fine chemical suppliers at low cost.

High crystallinity P_black_ was originally synthesized directly from P_red_/P_4_ by heating under pressure in the GPa range,^[1b]^ typically forming single millimeter‐sized crystals. Low pressure alternative syntheses are feasible through vapor crystal growth using a metal catalyst, such as AuSn/SnI_4_, creating gram‐scale P_black_ single crystals, over several days.^[17]^ These high quality P_black_ materials are available commercially and have underpinned research in phosphorene and its related nanomaterials, but the intrinsically small scale and lengthy synthesis leads to high costs, on the order of several hundred dollars for sub‐gram crystals; over 5 orders of magnitude more expensive per gram than P_red_.

To address the significant issues of cost and scale, there is ongoing work to improve the production of P_black_. Recent attempts to scale the P_black_ pressure synthesis through appropriation of ceramic processing techniques have been used to create high density nanocrystalline ‘ceramics’ with sub‐50 nm P_black_ domains at the 10 g scale, which could be exfoliated to smaller multilayer agglomerates through solvent assisted shear force exfoliation.^[18]^ Alternatively, P_black_ may be synthesized through ball‐milling of P_red_ under inert atmosphere,^[19]^ using the high local pressure and temperature to facilitate the phase transition to the thermodynamically stable, higher density P_black_ crystallites. Conversely, ball‐milling single‐crystal P_black_ leads to degradation from local bond shearing,^[20]^ forming amorphous P_red_ regions, indicating that ball‐milling is unlikely to provide a route to highly crystalline, microscale P_black_/phosphorene. By undertaking ball‐milling in a liquid, it is possible to directly synthesize small BPQDs (~5 nm) from P_red_, although ≥50 % of the P atoms are bound to oxygen.[^10^, ^21^]

**Figure 1:**
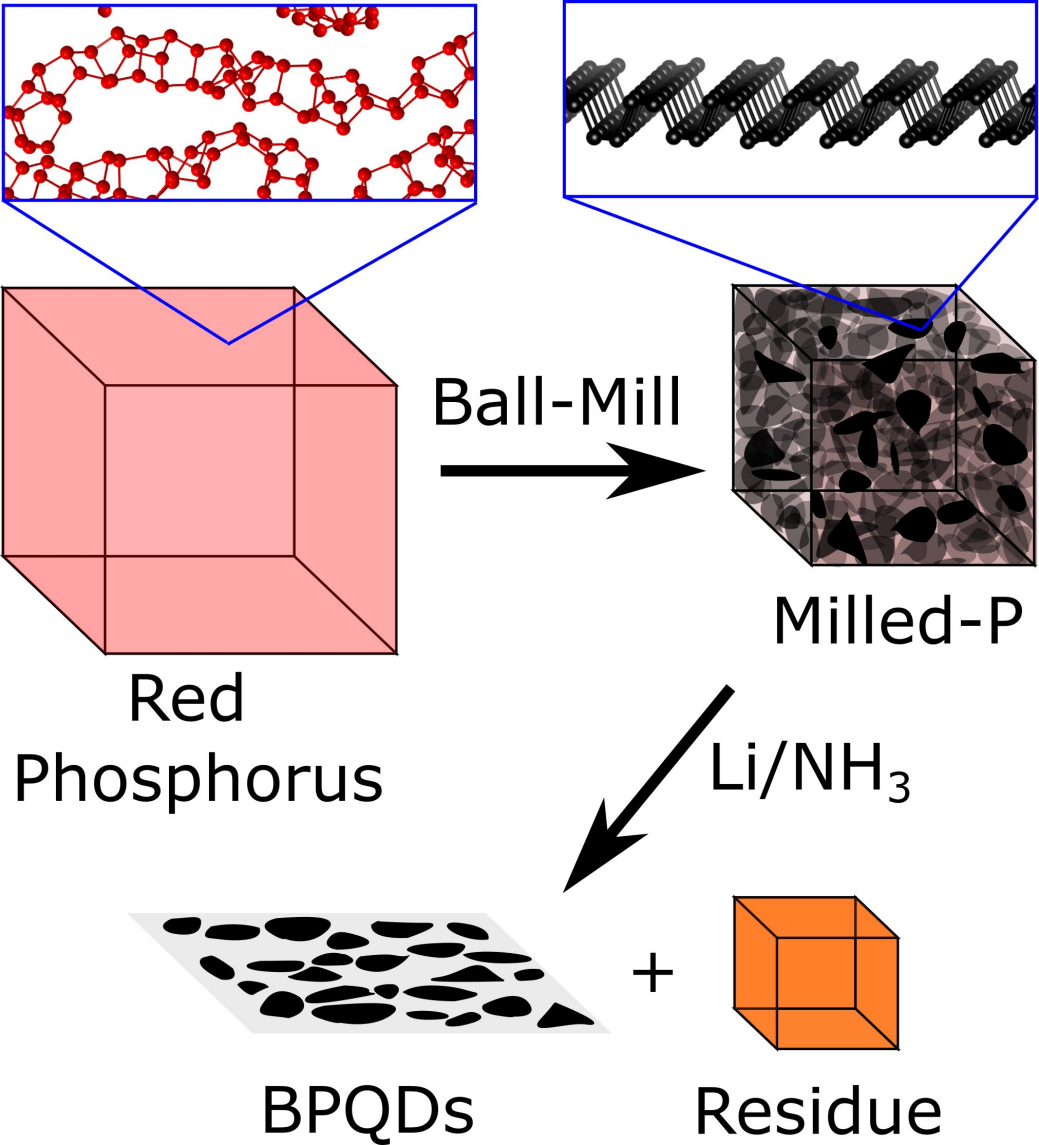
Schematic of the conversion of amorphous P_red_ to BPQDs via ball‐milling and reductive etching in lithium electride.

Here, to access scalable, low‐cost BPQDs with lower oxygen functionality, we use a two‐step process: firstly, dry ball‐milling of P_red_ to produce a nanocrystalline P_black_ material, and subsequently separating isolated BPQDs through reductive etching (Figure 1).

## Results and Discussion

**Figure 2:**
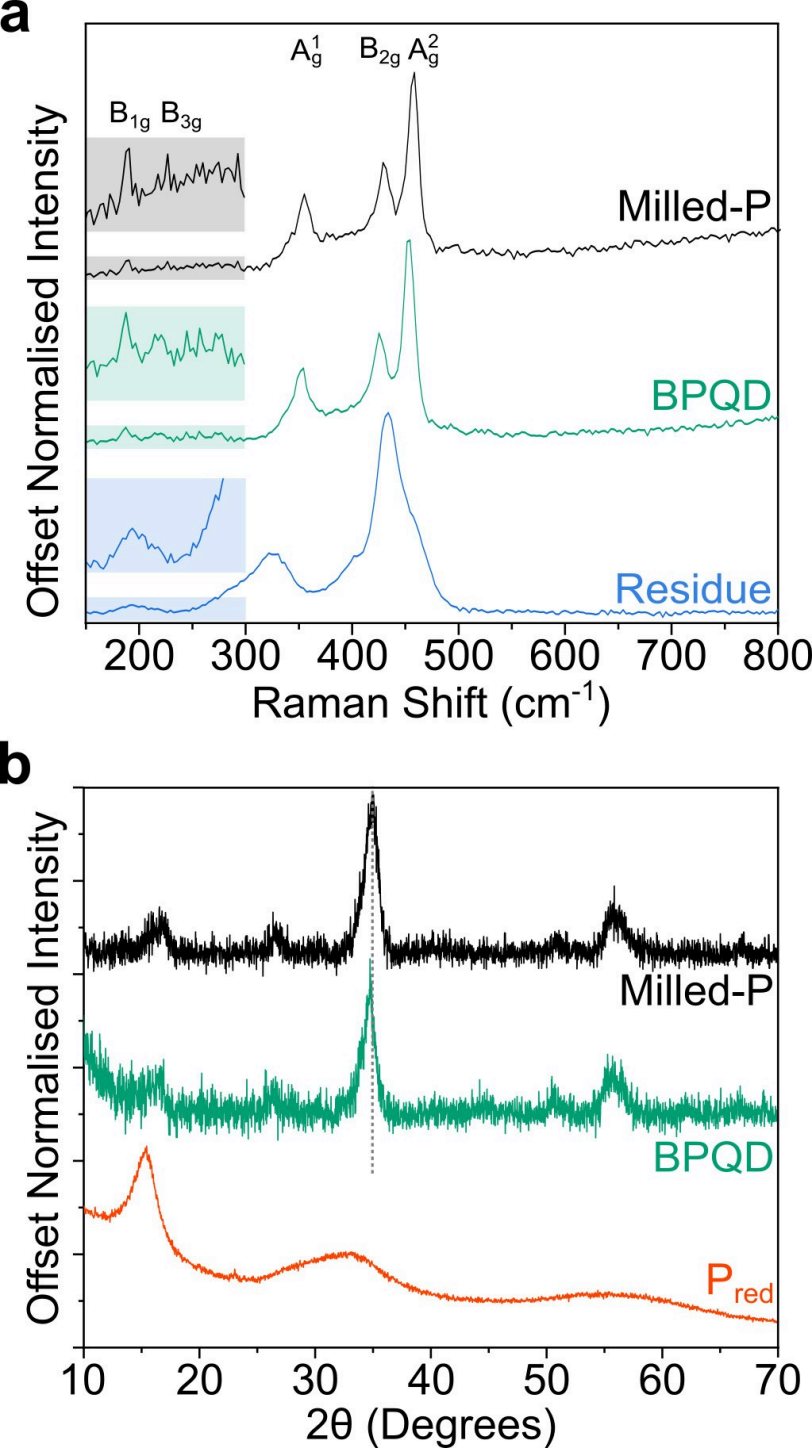
a) Raman spectra of Milled‐P, BPQDs, and Residue. Insets: magnified regions between 150–300 cm^−1^ to highlight the edge‐modes. Additional Raman data is provided in Supporting Information Figures S1 and S2. b) pXRD of as‐received P_red_, Milled‐P, and BPQD powder.

Black phosphorus powder was synthesized through dry ball‐milling of P_red_, producing a dark powder with a slight red coloring (which is shown to contain P_black_ domains but is named “Milled‐P” here to distinguish from pure macrocrystalline orthorhombic P_black_). The Raman spectra of Milled‐P uniformly showed the $A_{g}^{1}$ , $B_{2g}$ and $A_{g}^{2}$ modes of P_black_ with maxima at 354, 428, and 457 cm^−1^, respectively (Figure 2a), which is similar to other reports of P_red_ to P_black_ preparations via ball‐milling,[^19b^, ^22^] although significantly red‐shifted when compared to highly crystalline P_black_ (~10 cm^−1^).^[23]^ The variation may be attributed to residual strain from the ball‐milling process: comparable red‐shifting was observed by Karki et al.^[24]^ in few‐layer P_black_ under tensile strain. The raised baseline between the $A_{g}^{1}$ /$B_{2g}$ modes indicates the concurrent presence of P_red_ Raman modes^[22]^ within the sample (Supporting Information, Figure S1). Additionally, small peaks at 187 cm^−1^ and 219 cm^−1^ were seen, attributed to the $B_{1g}$ and $B_{3g}$ modes seen at P_black_ edges,^[23a]^ indicative of a large proportion of phosphorene edges within the Milled‐P material (Supporting Information, Section S1). The XRD pattern of Milled‐P was typical of previously produced ball‐milled P_red_ (Figure 2b), with reduction in diffuse scattering features characteristic of P_red_, and the emergence of P_black_ reflections,^[25]^ albeit significantly broader than single‐crystal P_black_.[^22b^, ^26^] The material could not be dispersed in amidic solvents with stirring or bath sonication.

As crystalline P_black_ is known to be more enthalpically stable^[18]^ (Δ*H*_f_=−21.8 kJ mol^−1^) and higher density^[27]^ (*ρ*P_red_≤2.33 g cm^−3^, *ρ*P_black_=2.69 g cm^−3^) than P_red_, previous literature has attributed the mechanism of conversion during ball‐milling to local compression that converts phosphorus to the orthorhombic crystalline P_black_ phase. The mechanism may be seen as analogous to P_black_ synthesis via compression of P_red_ under isotropic pressure, however, this latter route leads to macroscopic crystals and an absence of P_red_ modes in the Raman spectrum. The transient, uniaxial and highly localized high pressures in ball‐milling appear to synthesize orthorhombic P_black_ with a degree of long‐range disorder.^[22a]^ From the presence of P_red_ Raman modes and broadened XRD signal in our ball‐milled material, we suppose that Milled‐P consists predominantly of close‐packed P_black_ nanocrystallites, connected with less‐ordered grain boundaries and a fraction of amorphous P_red_.

To separate isolated crystalline black phosphorene domains from Milled‐P, it is necessary to remove the non‐crystalline P surrounding them. To this end, the difference in reductive stability between the allotropes may be exploited: P_red_ is known to decompose to molecular metal phosphides in highly reducing conditions,^[28]^ while P_black_ (nano)structures have been shown to reduce but retain their macroscopic atomic framework as nanomaterial anions.[^23b^, ^29^] By subjecting the Milled‐P to highly reducing conditions, the discrete black phosphorene domains would be anionically charged but remain intact, while other forms of phosphorus present (i. e., atoms along disordered grain boundaries and residual P_red_) would be etched away by conversion to small molecular phosphides, freeing the discrete nanocrystalline phosphorene domains. We note that highly reducing conditions can also eventually degrade black phosphorene‐based materials,^[30]^ so only a fraction of reductant versus phosphorus should be used. Similarly, low temperatures should be selected to maximize the reactivity difference between the disordered phosphorus's and black phosphorene.

Lithium electride ammonia solution was chosen as the reducing system of choice to etch disordered regions to free the crystalline black phosphorene component, due to its success in recovering phosphorene materials previously, and its low intrinsic temperature. Ammonia was condensed over an 8 : 1 molar ratio mixture of Milled‐P and lithium, and the solution immediately turned blue, indicative of the formation of dilute ammonia‐solvated electride but turned red overnight. After evaporation of the ammonia, a significant fraction of dark orange residue (hence termed “Residue”) formed as coffee rings around the edges of the flask, leaving a black powder at the bottom, shown to contain BPQDs (see below). The Residue is attributed to lithium phosphides, as seen previously as the product of P_red_ reduction^[28]^ and as trace impurities in P_black_ reduction during PNR synthesis[^29^, ^31^] and group 1 metal intercalation.^[23b]^

**Figure 3:**
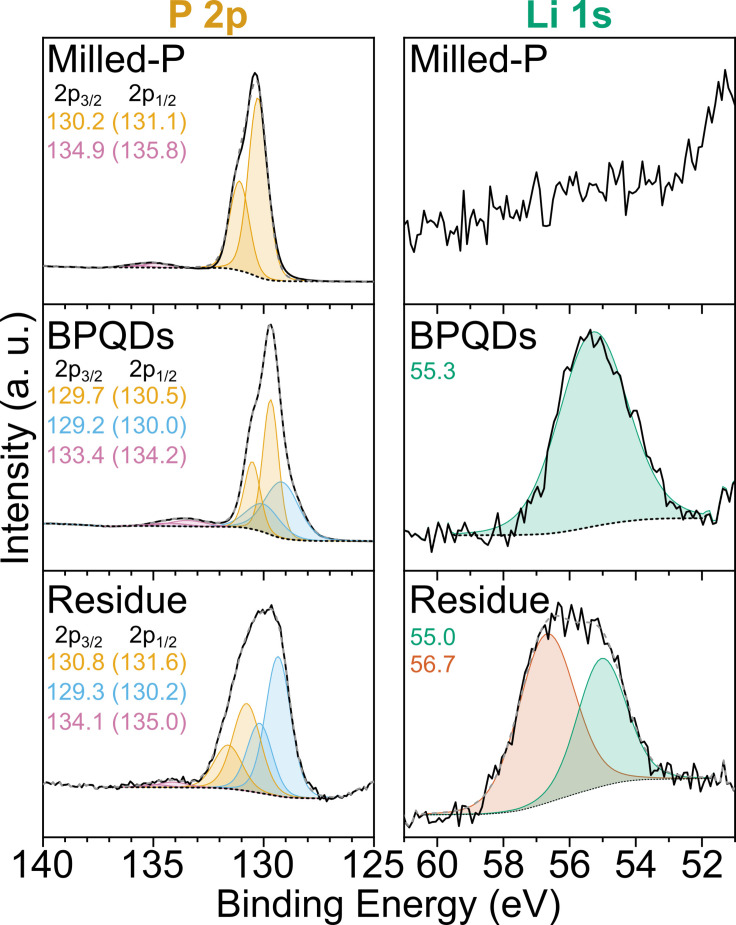
XPS spectra in P2p (left) and Li1s (right) regions. Fitted peak center values inset. N.B. Feature at ~51 eV is attributed to trace Mg impurity. Black solid ‐ Measured data; Coloured solid – Fitted components; Grey dashed – Sum of fit; Black dashed – Fitted background. Additional elemental/survey scans, and ratios are provided in Supporting Information Figures S3 and S4.

The pXRD of the BPQDs maintained the P_black_ reflections and the Raman spectra of the BPQDs show a small red‐shift of all three prominent modes (to $A_{g}^{1}$ ~352, $B_{2g}$ ~426, and $A_{g}^{2}$ ~453 cm^−1^) compared to Milled‐P, as has been seen for reduced phosphorene‐derived materials previously,^[23b]^ and the $B_{1g}$ mode is maintained. The added lithium is observable in the XPS (Figure 3) of BPQDs, assigned as Li^+^ (55.3 eV). The P2p signal is split, with the most intense peak slightly downshifted versus P_black_ (2p_3/2_ from 130.2 to 129.7 eV), with the subtle change attributed to slight delocalization of additional electron density across the phosphorene domains. A second, broader signal is seen at lower energy (129.2 eV) attributed to anionic P atoms, consistent with previous lithium phosphide literature values.^[32]^ In contrast to other P_red_‐derived BPQD syntheses, there is no rise in the P−O region (>133 eV).

The BPQD powder was spontaneously soluble in dimethylacetamide (DMAc), dimethylformamide, and N‐methyl pyrrolidine, forming a dark red cloudy suspension without agitation over several hours, which was maintained after centrifugation at ~100 *g* for 30 min. The decanted DMAc solution is used for subsequent characterization. The solution is air sensitive, with a precipitate forming over several minutes after air exposure, attributed to loss of the stabilizing charge akin to anionic nanocarbon solutions, although degradation of phosphorene is a possible alternative.^[33]^

**Figure 4:**
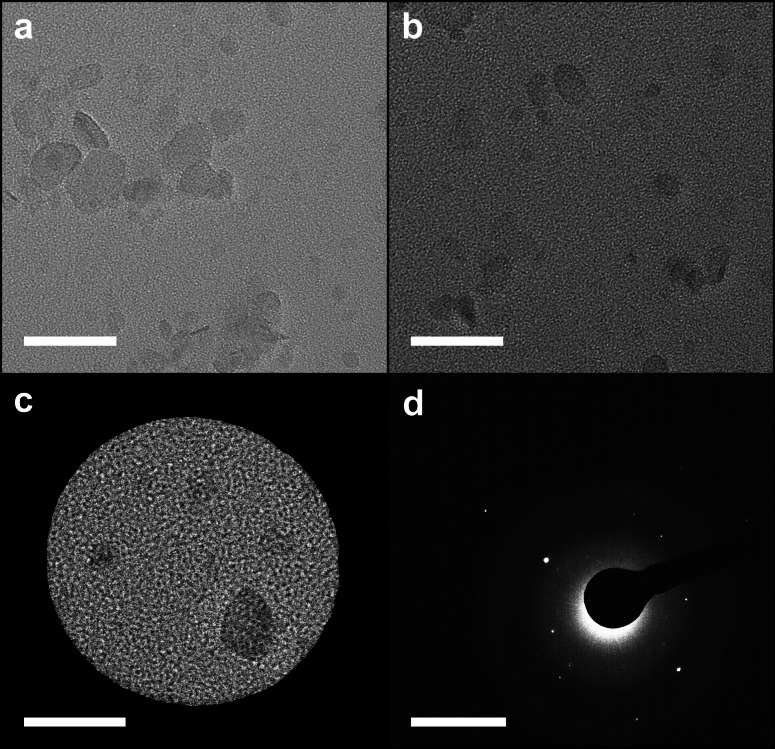
TEM of BPQDs. a–c) TEM micrographs, scale bar 50 nm. d) SAED of (c), scale bar=1 nm^−1^. Further TEM micrographs are provided in Supporting Information Figure S5.

**Figure 5:**
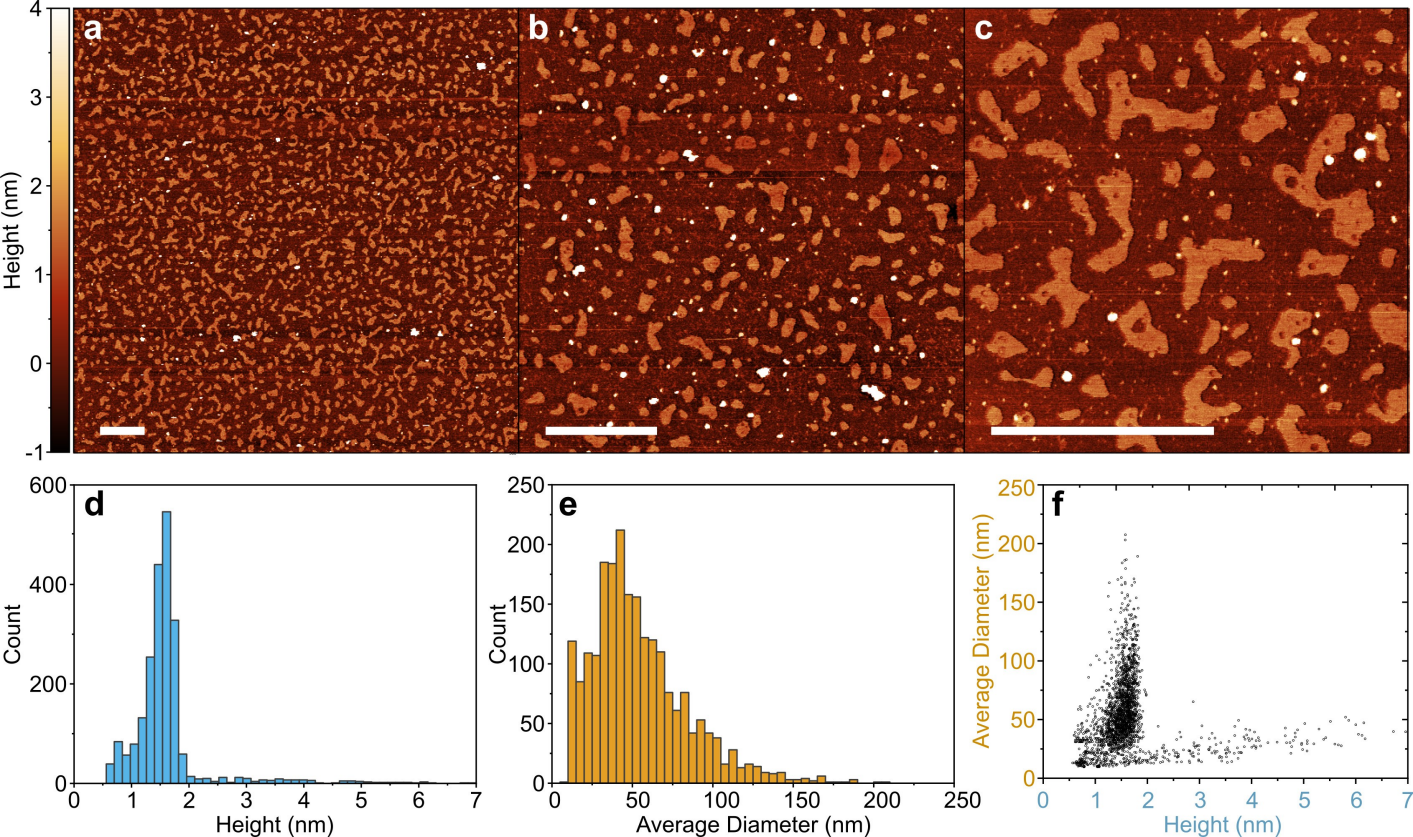
AFM of BPQDs. a–c) AFM micrographs of BPQD on mica; scale bars 500 nm. d) Height distribution (50 bins, 0–7 nm). e) Average diameter distribution (50 bins, 0–250 nm). f) Height versus average diameter. Facet analysis for size distribution provided in Supporting Information Section S2.

Transmission electron microscopy (TEM) of the drop‐cast solution shows that the material is formed primarily of BPQDs with lateral dimensions of approximately 20–100 nm (Figure 4). Selected area electron diffraction (SAED) of an individual ~25 nm BPQD shows the crystalline domains of the produced material. Atomic force microscopy (AFM) of the spontaneous solution drop‐cast on mica and extensively dried, is in broad agreement with the TEM with the average length (calculated from the square root of the measured area) centering around 50 nm (Figure 5e). The BPQD heights measured by AFM are centered at ~1.6 nm with 92 % of all species having heights <2 nm. High quality 2D black phosphorene grown by pulsed laser deposition on mica^[34]^ has previously been measured to have a height of 1.1 nm, indicating that the species here are primarily monolayer, but defective, leading to buckling of the layers. The measured height is also lower than that of other phosphorene quantum dots:[^12^, ^13^] violet phosphorus quantum dots are 1.8 nm, and fibrous phosphorus quantum dots, 2.7 nm, supporting the assignment as BPQDs.

The defectiveness can also be seen clearly from some of the larger BPQDs exhibiting holes in the structure (Figure 5c). Smaller holes/vacancies may be present throughout the samples but are outside the resolution of the AFM. While the more prevalent smaller BPQDs show oblong geometries, the largest flakes are all monolayer and appear to consist of multiple overlapping oblong features, which we attribute to the milling nature of the synthesis. Local compression during milling leads to formation of ~10–50 nm phosphorene domains, where compression of adjacent phosphorene domains leads to fusing into larger phosphorene species.

**Figure 6:**
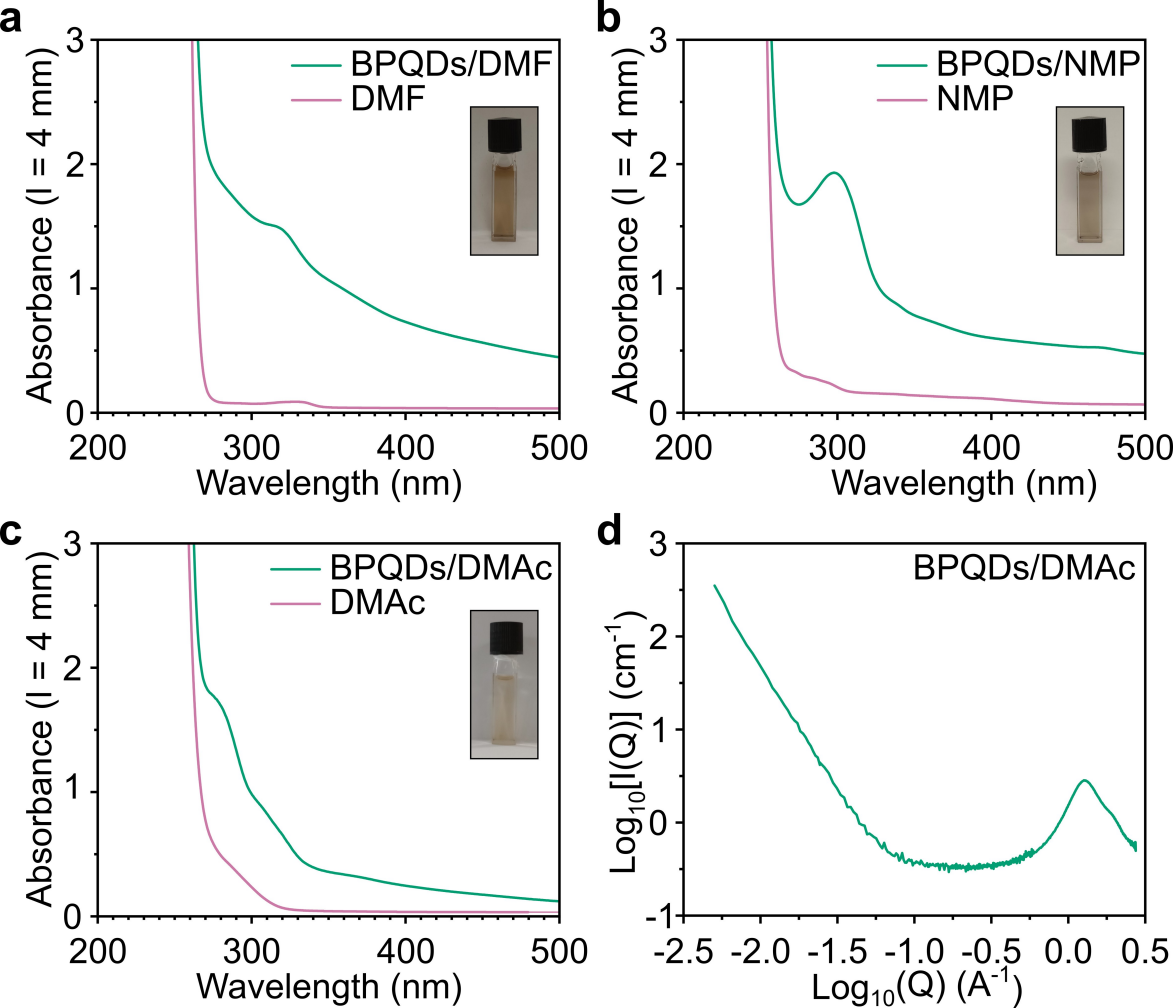
Properties of BPQD solution. a–c) UV‐vis spectrum of dissolved BPQDs (initial loading ~1 mg mL^−1^) and neat solvent of a) DMF, b) NMP and c) DMAc, with digital photographs inset. Full UV‐vis ranges provided in Supporting Information Figure S6(a). d) SAXS of DMAc BPQD solution, further information provided in Supporting Information Figure S7.

The BPQD solution has three absorption modes in the UV‐visible range (Figure 6a–c): a strong peak around 275 nm (overlapping with the strong solvent absorption <260 nm) a mode around 325 nm and a weak peak around 375 nm (best seen in the derivative, Supporting Information Figure S6b) – the latter two peaks mimic similar optical absorbances seen in PNRs.^[29]^ To better understand the nature of solvated material, the Small Angle X‐ray Scattering (SAXS) measurements of the solution was taken over a Q‐range of 0.0004–2.75 Å^−1^. For dispersed 2D platelets, scattering intensity in the low‐Q range is proportional to *Q* ^−D^, where *D*~2 for fully dispersed 2D platelets, while 3D species typically have *D* between 3 and 4.^[35]^ Fitting our data in the *Q*<0.02 Å region provided an intermediate D of 2.98, indicating high aspect ratio nanoscale 3D species, which broadly agrees with the large fraction of low diameter quasi‐2D BPQDs.

**Figure 7:**
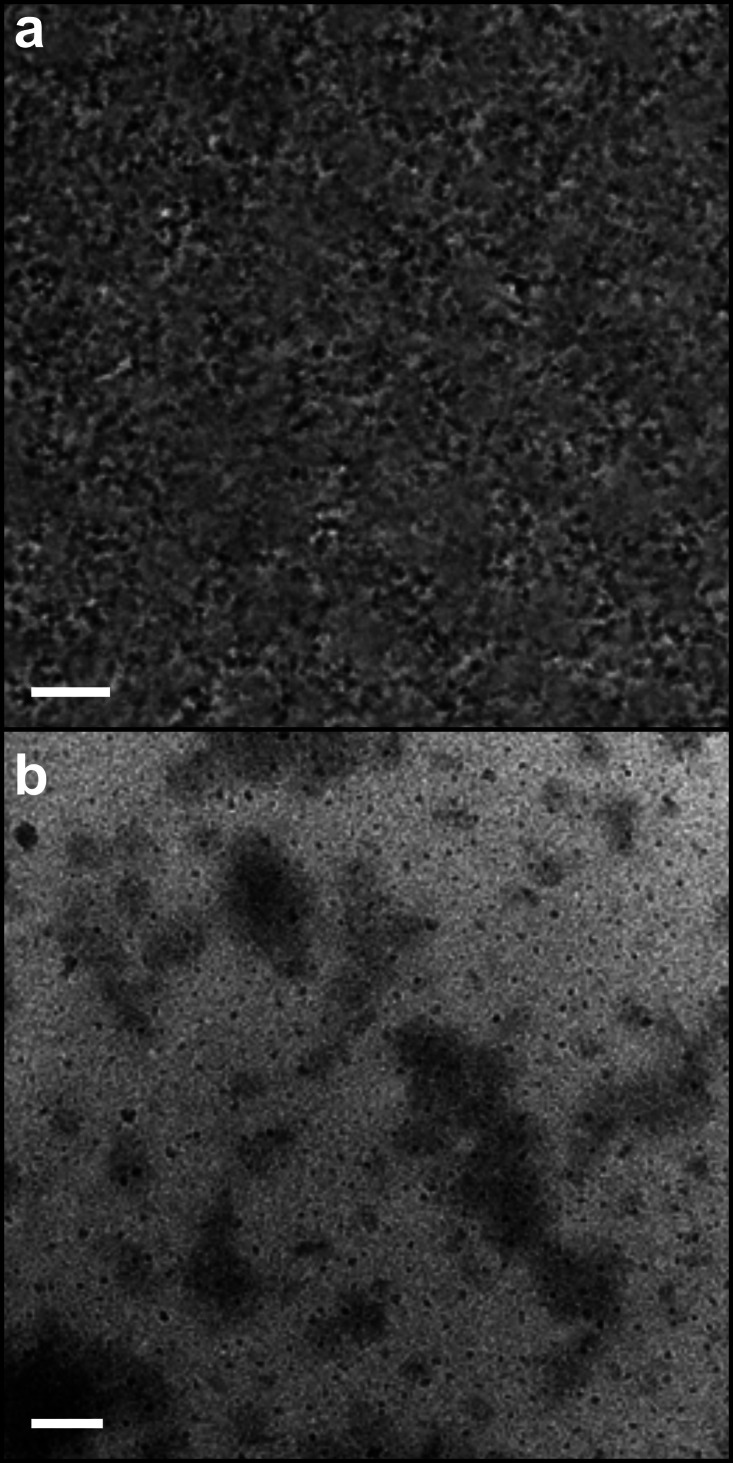
LP‐TEM of BPQD DMAc solution. a) A single frame from the liquid cell movie of the particle dynamics, denoised using the noise‐to‐noise algorithm in Topaz, scale bar 1 μm. b) In situ‐dried specimen, scale bar 100 nm. Further data and explanation of the liquid TEM processing in the Supporting Information, Section S4.

Liquid Phase TEM (LP‐TEM) was used to verify the presence and mobility of the 3D species in solution. LP‐TEM intrinsically has high‐noise derived from the additional electron scatter from the solvent, which is particularly problematic when imaging atomically thin solutes. While graphene has been used for nanofluidic chip windows,^[36]^ to the authors’ knowledge, there are no previously reported measurements of a liquid‐phase (quasi‐)2D material in LP‐TEM. Here, nanofluidic chips with 60 nm height channels were used for the clearest possible imaging of the atomically thin particles in a liquid environment. The TEM observations were recorded at relatively low magnification to minimize both electron beam damage effects in the liquid and formation of gas bubbles. Despite optimizing the imaging conditions, the particles are still low contrast and are only present in a few frames at a time while free to diffuse in the liquid. However, BPQDs can be seen across multiple frames when they are slowed by interactions with the edge or top of the nanochannel (Figure 7a). To improve our confidence in the presence of the particles within the liquid during these observations, the sample was dried in situ, allowing remaining particles to be imaged to compare and contrast with the liquid observations (Figure 7b).

To confirm the mechanism of formation of BPQDs from Milled‐P, the Residue was characterized. The Raman spectrum (Figure 2a) showed significantly broader, downshifted features versus phosphorene, and appears to maintain the $B_{1g}$ mode. Similar broadening has been seen for highly charged graphene^[37]^ and is indicative of some fraction of phosphorus having anionic P_black_ character. The XPS (Figure 3) also confirms the presence of lithium phosphide formation as expected from reaction between lithium electride and both P_red_ regions/disordered phosphorene grain boundaries of Milled‐P. The phosphide peak is present as the most intense component (P2p_3/2_ 129.3 eV) and an excess of lithium over phosphorus is detected (Li_1.6_P). Concurrently, unlike the BPQDs, the more neutral P signal (130.8 eV) is not downshifted versus the P(0) detected in the Milled‐P sample, implying that delocalization of negative charge is not present, as expected where P_red_ is the dominant phase. The Li 1s signal splits, with both peaks in the Li^+^ range, tentatively attributed to formation of Li_2_O from the more highly charged and reactive phosphide species in the Residue sample combined with the imperfect air‐free loading of the XPS samples. The etching of disordered phosphorus can also be inferred from the holes in the larger BPQDs (Figure 5), which presumably were previously filled with reduction‐unstable amorphous phosphorus.

## Conclusions

In conclusion, BPQDs may be synthesized from low‐cost red phosphorus through ball‐milling to create nanoscale phosphorene domains surrounded by amorphous red phosphorus. By etching the P_red_ component with lithium ammonia solution, high crystallinity phosphorene quantum dots with 10–50 nm diameters are created. The produced nanomaterials are spontaneously soluble as monolayers in tertiary amide solvents, providing a route to liquid‐phase assembly processes such as printing or spin‐coating. The synthesis is simple, intrinsically scalable, and uses low‐cost precursors (P_red_ and Li), while requiring equipment widely available in research laboratories (gas handling, ball‐mill), opening the way for wider use of BPQDs in research and industry.

## Experimental Section

**Materials**: P_red_ (98.9 %) was purchased from Alfa Aesar. Lithium (99.9 %, rod), ammonia (99.999 %), dimethylacetamide (99.8 % anhydrous), dimethylformamide (99.8 % anhydrous), and N‐methyl pyrrolidone (99.5 %, anhydrous) were purchased from Sigma Aldrich (UK). Ammonia was purified through condensation over excess Li metal prior to use, and amidic solvents was dried by soaking over activated 4 Å molecular sieves for >7 days prior to use.

**Milled‐P Synthesis**: Black phosphorus powder (Milled‐P) was prepared by milling 2.4 g of red phosphorus in a 250 mL stainless steel jar using a Fritsch Pulverisette 5 planetary ball mill under an Ar atmosphere for 25 h (200 kPa in excess of atmospheric pressure). Four stainless steel balls, each 25.4 mm in diameter, were used for milling. The ball‐to‐powder ratio was maintained at 110 : 1, and the milling was carried out at a rotation speed of 250 rpm. The as‐milled powders were unloaded from the jar inside an Ar glove box.

**BPQD Synthesis**: In an Ar glovebox, Milled‐P (typically 100 mg) was placed in a glass tube fitted with a glass‐metal transition alongside freshly cut lithium (0.125 mol. equiv. vs P). The tube was fitted with a steel Swagelok valve, sealed, and removed from the glovebox. The tube was then evacuated (~10^−7^ mbar) and submerged in a −50 °C cooling bath (isopropyl alcohol with chiller). Ammonia was condensed into the flask until both lithium and Milled‐P were covered, with the solution quickly turning dark blue (i. e., lithium electride). The solution was left for 16 h, after which the liquid had turned dark orange. Ammonia was then evaporated to leave a black powder at the bottom, surrounded by thick, orange coffee‐rings (Residue). The black powder of BPQDs was carefully removed and set aside, and the residue was scraped off the sidewalls. N.B., ammonia is a toxic gas and must be handled with care – guidance and advice for the condensation and removal is provided in Supporting Information Section S3. Metal phosphide contaminated glassware was rinsed with bleach to oxidize to acids in a fumehood before cleaning.

**X‐ray fluorescence spectroscopy**: XPS was undertaken on a K‐Alpha instrument (Thermo Scientific) equipped with monochromatic Al *K*_α_ radiation (h ν=1486.6 eV) as incident X‐ray source. Samples were adhered to a substrate with indium foil and loaded air‐free using a vacuum transfer vessel (Thermo Scientific 831‐57‐100‐2). Data were fitted with CasaXPS (v2.3.19PR1.0) with Shirley background and 2p spin orbit coupled peaks constrained to equivalent FWHM, at 1 : 2 integral ratio, and fixed peak center separations (P=0.87 eV; Si=0.61 eV). Data was calibrated against C1s of adventitious carbon (284.8 eV).

**Transmission electron microscopy**: TEM was performed on a JEOL 2200 FS, with an accelerating voltage of 200 keV. Dry samples were prepared in an Ar glovebox by dropping ~30 μL of centrifuged solution directly onto a TEM grid (holey carbon on Au, Agar Scientific) placed on a cellulose filter paper (Whatmann) and left to dry overnight. Samples were transported to the TEM under Ar, and exposed to air during loading onto the TEM specimen holder and into the TEM machine. For liquid phase TEM, a Nanofluidic chip (Insight Chips) was used, consisting of 9.9 nm Si_3_N_4_ and 3 nm Al_2_O_3_ windows, with 60 nm path length. The height was selected to minimize solvent scatter but may bias against imaging of larger BPQD species. Liquid channels are cut into multiple dumbbells, stemming from a central path, allowing liquid in the dumbbell ends to be imaged. In an Ar glovebox, a clean cannula was used to pierce the inlet and outlet slots on the chip and ~10 μL of BPQD DMAc solution (10× diluted from the initial 1 mg mL^−1^ loaded solution) was pipetted on the inlet, quickly being intaken by capillary forces. The sample was transferred to the TEM under Ar, and quickly loaded into an Insight Chips Liquid Phase TEM holder and immediately transferred to the TEM. Data were recorded using a Gatan K2‐IS detector to minimize the electron flux exposure required to observe an image, additionally an in‐column energy filter with a 20 eV slit was used to reduce the background from inelastic scattering of the liquid, giving the images an enhanced contrast. Drying was performed by rupturing the window of a dumbbell using the electron beam. Detailed information on image post‐processing provided in Supporting Information, Section S4.

**Atomic force microscopy**: AFM was performed on a Bruker Dimension Icon in peak force tapping mode, using MSNL‐10 (Bruker) probes. Samples were prepared in an Ar glovebox through diluting decanted BPQD solutions by 100× with anhydrous DMAc, and dropcasting 10 μL on freshly cleaved mica (Agar Scientific, UK, 25×25 mm) and drying overnight in the glovebox. Samples were then transferred to a glass tube fitted with a Swagelok valve and placed under dynamic vacuum (~10^−7^ mbar, Pfeiffer Vacuum HiCube) and heated to 100 °C for 48 h to remove residual solvent. Samples were cooled and returned to the glovebox and transferred under Ar to the AFM and exposed to air immediately before imaging. Data processing of AFM is described in Section S3.

**Raman spectroscopy**: Raman Spectroscopy was undertaken on a Renishaw inVia Raman Spectrometer, fitted with 488 nm laser. Potentially air sensitive samples (residue, BPQDs) were sealed in glass capillaries stoppered with wax under Ar for point‐spectra, and mapped samples were measured over a 200×200 μm square array with 20 μm separation (N=121), inside a custom‐made Swagelok cell, sealed under Ar. Pre‐milled P_red_ data for comparison purposes were collected separately, as the sample was located at Australian National University. Raman spectroscopy was performed using a Horiba Labram system equipped with confocal optics, a 532 nm diode‐pumped solid‐state (DPSS) laser, and a Si detector. Milled‐P was also measured under the same conditions for comparison to eliminate dispersion effects in comparing to the 488 nm measurements in the main text.

**UV‐vis spectroscopy**: UV‐vis spectra were measured using a Perkin Elmer Lambda 950 spectrometer. The BPQD in DMAc solution was diluted 10× with fresh anhydrous DMAc prior to measurement (and associated digital photograph) and placed in a screw‐cap 4 mm pathlength quartz cuvette (Hellma), sealed in a glovebox with PTFE tape inside the thread.

**Powder X‐ray diffraction**: pXRD of Milled‐P and BPQDs were recorded on a Bruker D2 phaser, with samples sealed in a glovebox within a PMMA dome fitted with a rubber O‐ring. Background subtraction was performed using DIFFRAC.EVA (Bruker). Pre‐milled P_red_ data for comparison purposes were collected separately, as the sample was located at Australian National University. X‐ray diffraction (XRD) pattern was collected using a PANalytical Empryrean instrument equipped with Cu *K*_α_ radiation (*λ*=1.54181 Å). The pattern was obtained using a step size of 0.026° and step time of 400 s.

**Small angle X‐ray scattering**: SAXS was measured with a SAXSLAB Ganesha 300XL, with as‐dissolved BPQD DMAc solution/anhydrous DMAc in borosilica 1 mm capillaries sealed with wax in a glovebox. The SAXS was calibrated against an AgBeh standard, and SAXS, MAXS and WAXS were measured for 1000, 300 and 100 s, respectively under <10^−2^ mbar vacuum.

## Supporting Information

Additional references cited within the Supporting Information.^[38]^

## Author Contributions

Experimental design and concepts were designed by AJC, CAH, & AG. Sample synthesis was performed by TR & AJC. Raman was performed by AJC, RRCS, AAW, & HTN. XPS was performed by RRCS & AJC. AFM was performed by AJC and ES. pXRD was performed by CDM & TR. SAXS was performed by CDM. UV‐vis were performed by AJC & RAI. (LP)TEM was performed by AS. Manuscript was written primarily by RRCS and AJC with contributions from all authors.

## Conflict of interest

The authors declare no conflict of interest.

1

## Supporting information

As a service to our authors and readers, this journal provides supporting information supplied by the authors. Such materials are peer reviewed and may be re‐organized for online delivery, but are not copy‐edited or typeset. Technical support issues arising from supporting information (other than missing files) should be addressed to the authors.

Supporting Information

## Data Availability

The data that support the findings of this study are available in the supplementary material of this article.
