# Supplementary material for: Synthesis of Black Phosphorene Quantum Dots from Red Phosphorus
Source: Chemistry. 2023 Sep 7;29(55):e202301232. doi: 10.1002/chem.202301232 (PMC10947263; doi:10.1002/chem.202301232)
Supplement: Supplementary file 1 — Supporting Information [file CHEM-29-0-s001.pdf]

Chemistry–A European Journal

Supporting Information

Synthesis of Black Phosphorene Quantum Dots from Red Phosphorus

Rebecca R. C. Shutt, Thrinathreddy Ramireddy, Evgenios Stylianidis, Camilla Di Mino, Rebecca A. Ingle, Gabriel Ing, Ary A. Wibowo, Hieu T. Nguyen, Christopher A. Howard, Alexey M. Glushenkov, Andrew Stewart,* and Adam J. Clancy*

Section S1. Additional Data

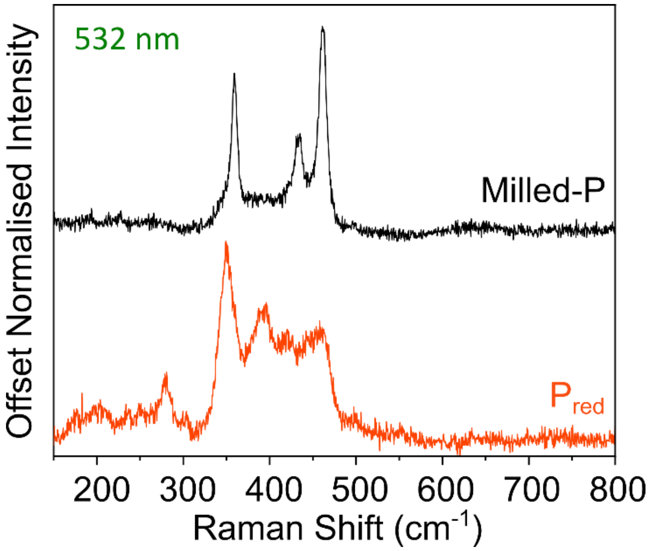

***Figure S1**. Raman spectra of Milled-P and precursor P_{red} recorded with 532 nm excitation. Data recorded separately at Australian National University compared to data in main text on Milled-P, BPQDs, and Residue which were recorded at UCL using 488 nm excitation (Main text, Fig. 2a).*

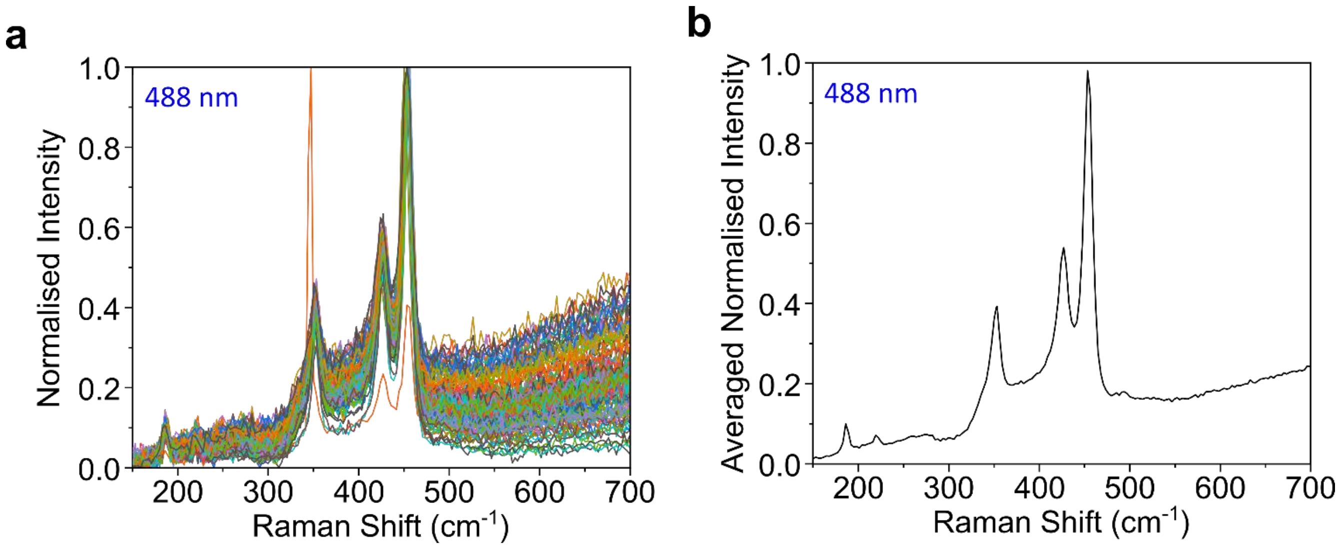

***Figure S2.** (a) Normalised Raman point spectra of Milled-P (488 nm excitation) taken in an 11 x 11 square array with 10 µm separation between points (N = 121), showing consistent presence of B_{1g} and B_{3g} modes associated with phosphorene edge states. (b) Average of data Fig. S2a for clarity.*

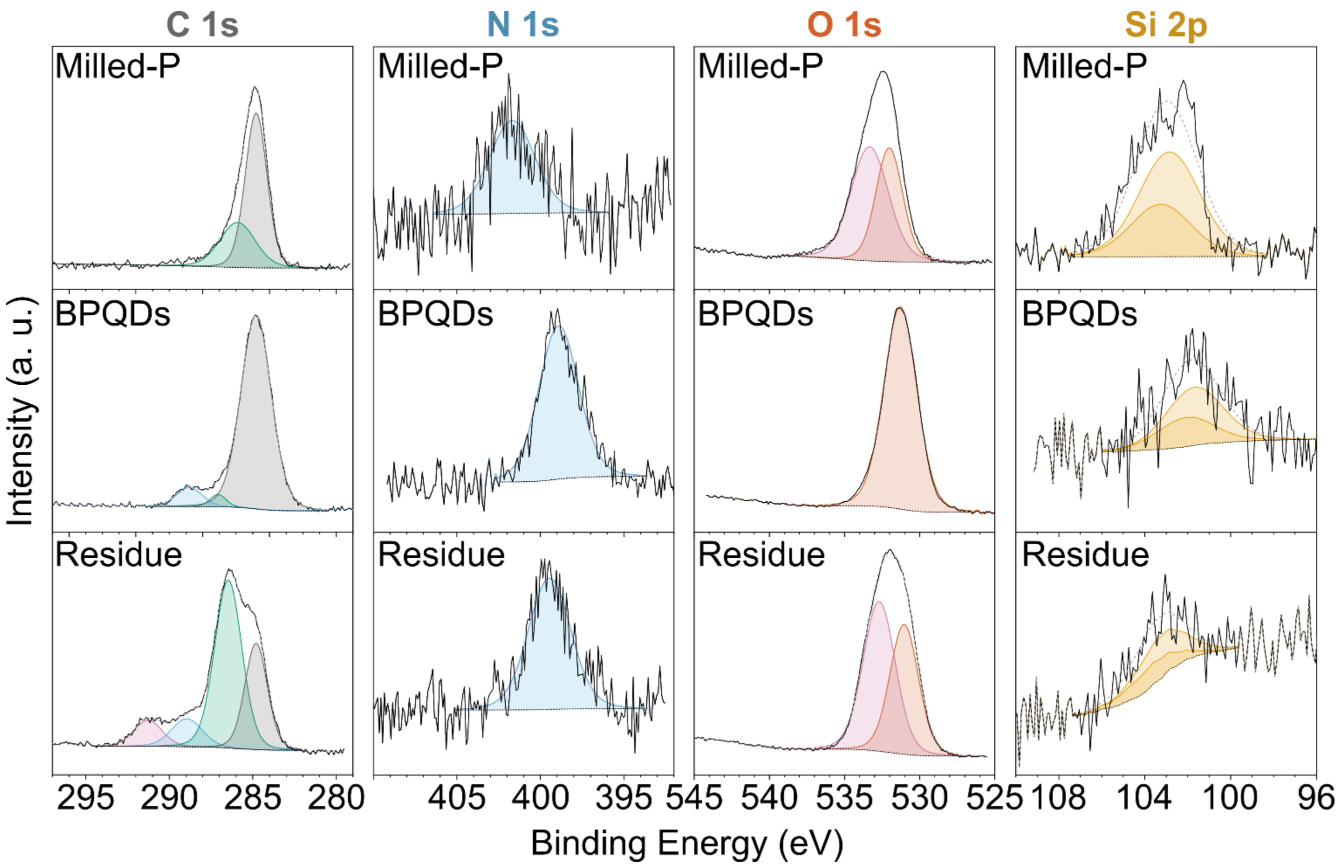

Figure S3. *(left to right) C 1s, N 1s, O 1s and Si 2p XPS spectra for Milled-P, BPQDs, and Residue. Black solid - Measured data; Coloured solid – Fitted components; Grey dashed – Sum of fit; Black dashed – Fitted background.*

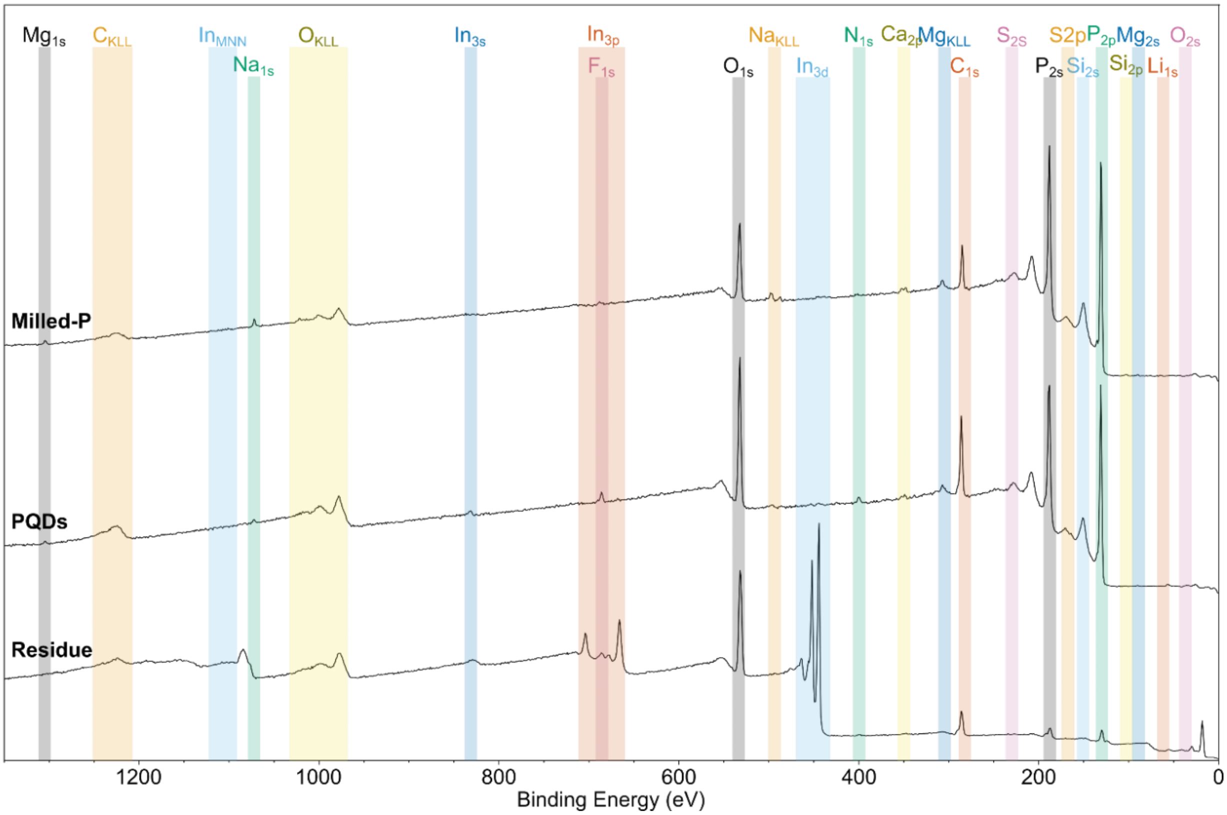

Figure S4. *Survey XPS scans of each sample. Regions for various elemental core-shell emissions and Auger transitions detected are indicated by shaded colours.*

Table S1. *Elemental ratios extracted from XPS spectra of Milled-P, BPQDs, and Residue.*

Milled-P

Element	***%***	***Ratio vs P***
C	17.25	0.26
Li	0.00	0.00
N	0.45	0.01
O	15.11	0.22
P	67.19	1.00

BPQDs

Element	***%***	***Ratio vs P***	***Ratio vs Li***
C	17.86	0.53	0.94
Li	19.01	0.56	1.00
N	1.67	0.05	0.09
O	27.57	0.81	1.45
P	33.89	1.00	1.78

Residue

Element	***%***	***Ratio vs P***	***Ratio vs Li***
C	21.18	1.27	0.81
Li	26.07	1.56	1.00
N	1.06	0.06	0.04
O	35.00	2.10	1.34
P	16.70	1.00	0.64

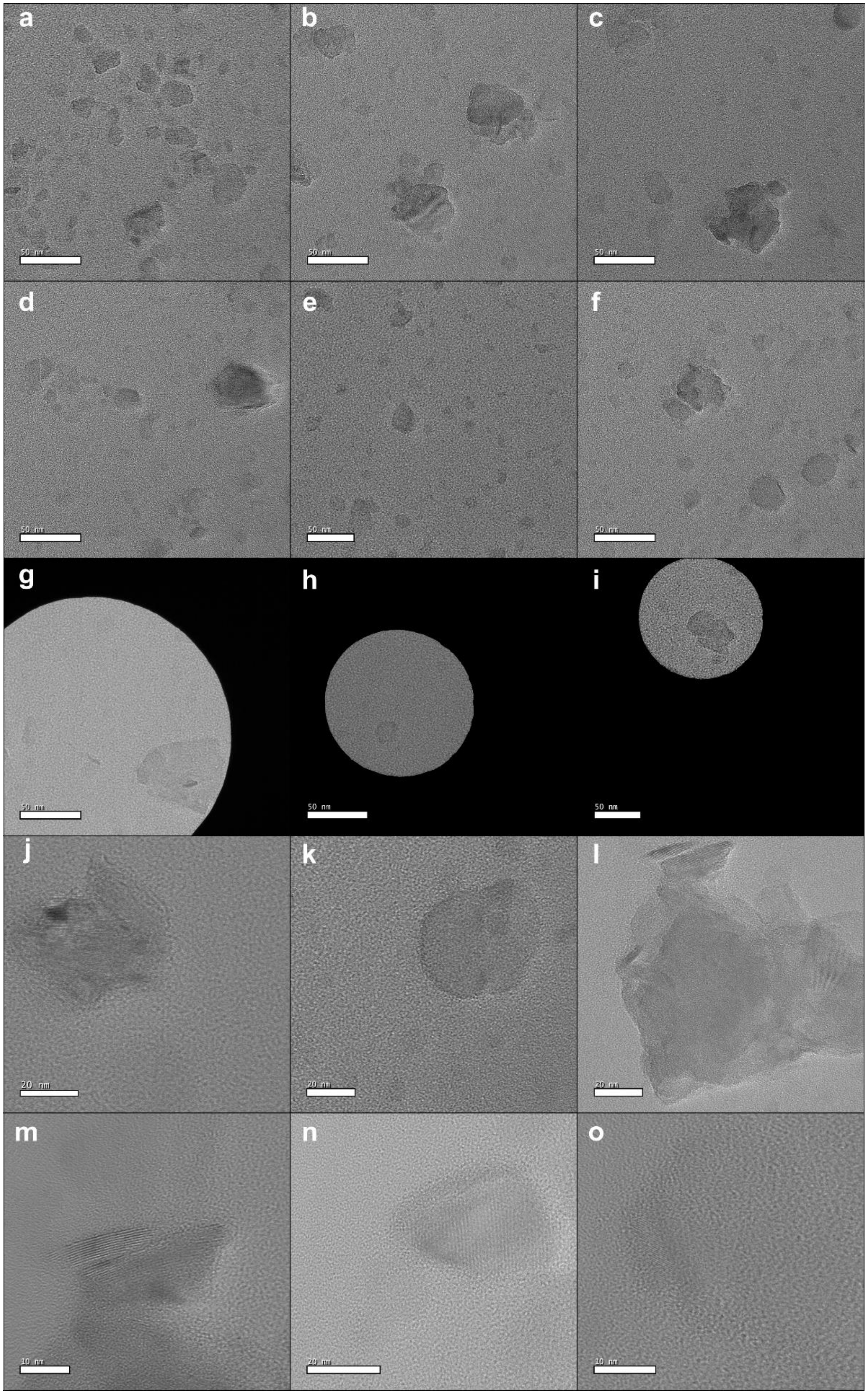

Figure S5. *Additional BPQD TEM micrographs. Scalebars = (a–i) 50 nm, (j–l) 20 nm, (m–o) 10 nm.*

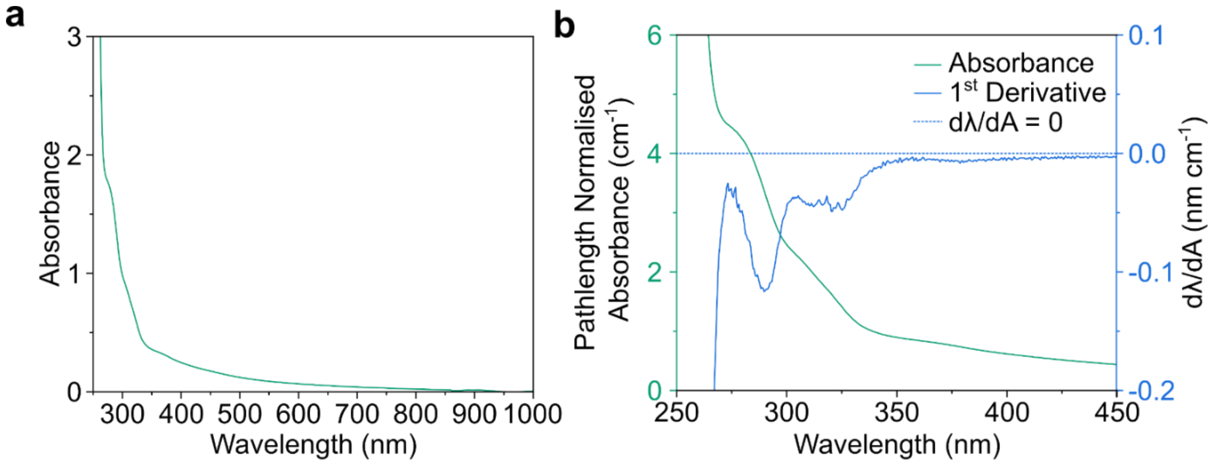

Figure S6. *Further optical spectra of BPQDs. (a) UV-vis spectrum of BPQDs in DMAc (10x diluted with DMAc from spontaneously dissolved solution) across the full measured wavelength range. (b) UV-vis spectrum from 250 nm – 450 nm, with 1st derivative, to better highlight the weak peak centred around 375 nm.*

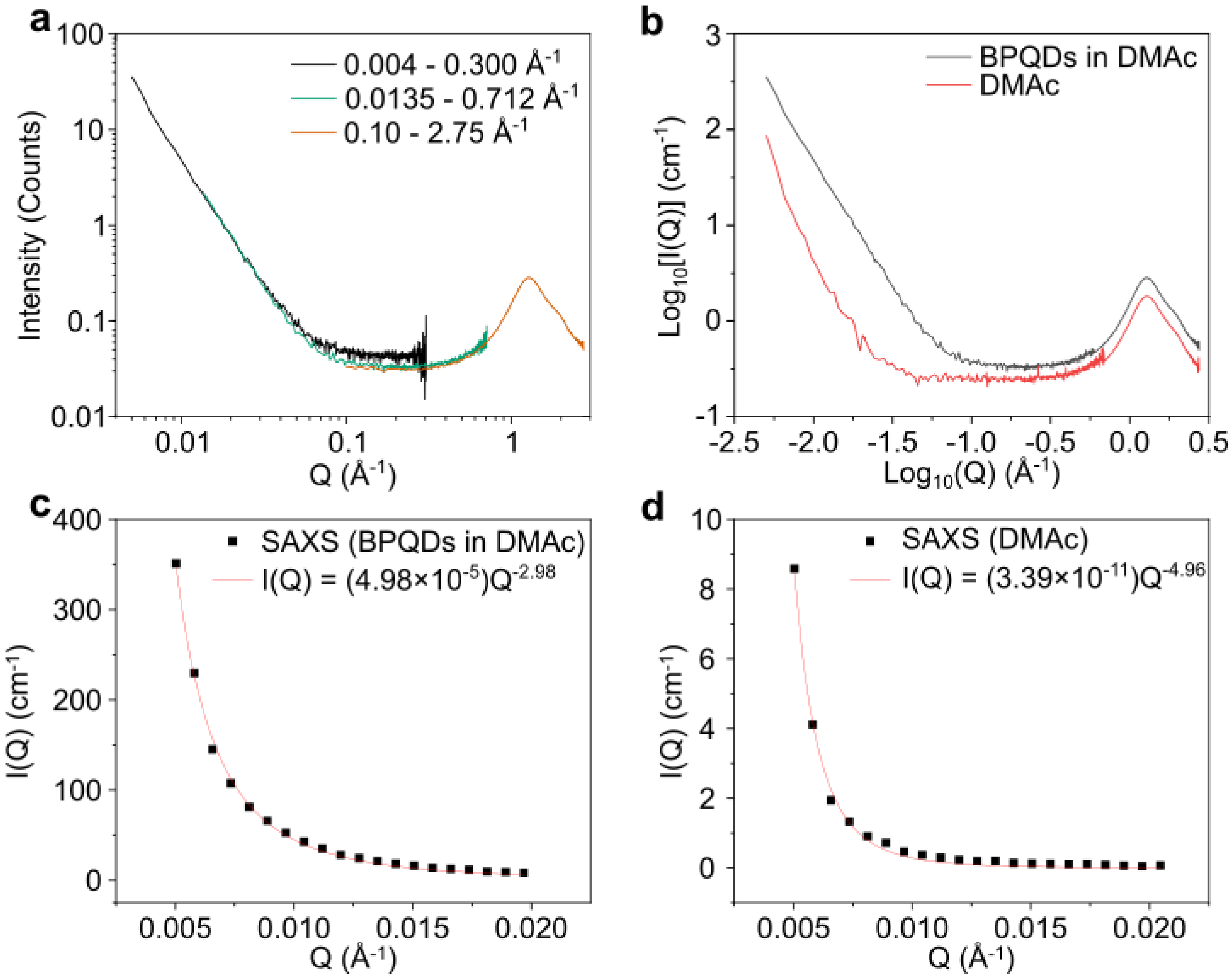

Figure S7. *Small Angle Xray Scattering Data. (a) Log-log plot of full non-truncated data for SAXS (0.004-0.3 Å^{-1}), MAXS (0.0155 - 0.712 Å^{-1}), and WAXS (0.1 – 2.75 Å^{-1}) scans on BPQD solution in DMAc dispersed at 1 mg mL^{-1} in 0.1 cm pathlength quartz capillary. (b) Truncated SAXS plots of BPQD solution (as plotted in main text, Fig. 6c) and pure DMAc solvent. (c,d) SAXS data at Q < 0.02 Å for BPQD solution and pure DMAc fitted to Eq. S1.*

$$I(Q) = Ae^{-DQ} \quad \text{Eq. S1}$$

Section S2. Supplementary AFM

Data Processing: AFM data was processed using Gwyddion (v2.61). The data background was levelled using the polynomial tool and the background silica was manually set to zero, with the z-scale set between -1 and 4 nm. For BPQD dimensional analysis, grains selected by a height threshold of 0.5 nm, and filtered to remove grains with < 10 pixels and grains at the micrograph edge. In total, N = 2195 grains were used for analysis. Micrographs with the grain masks used are provided below. Data was extracted as grain surface area and median height. Average diameters (in nm) were calculated as the square root of the surface areas (in nm^2).

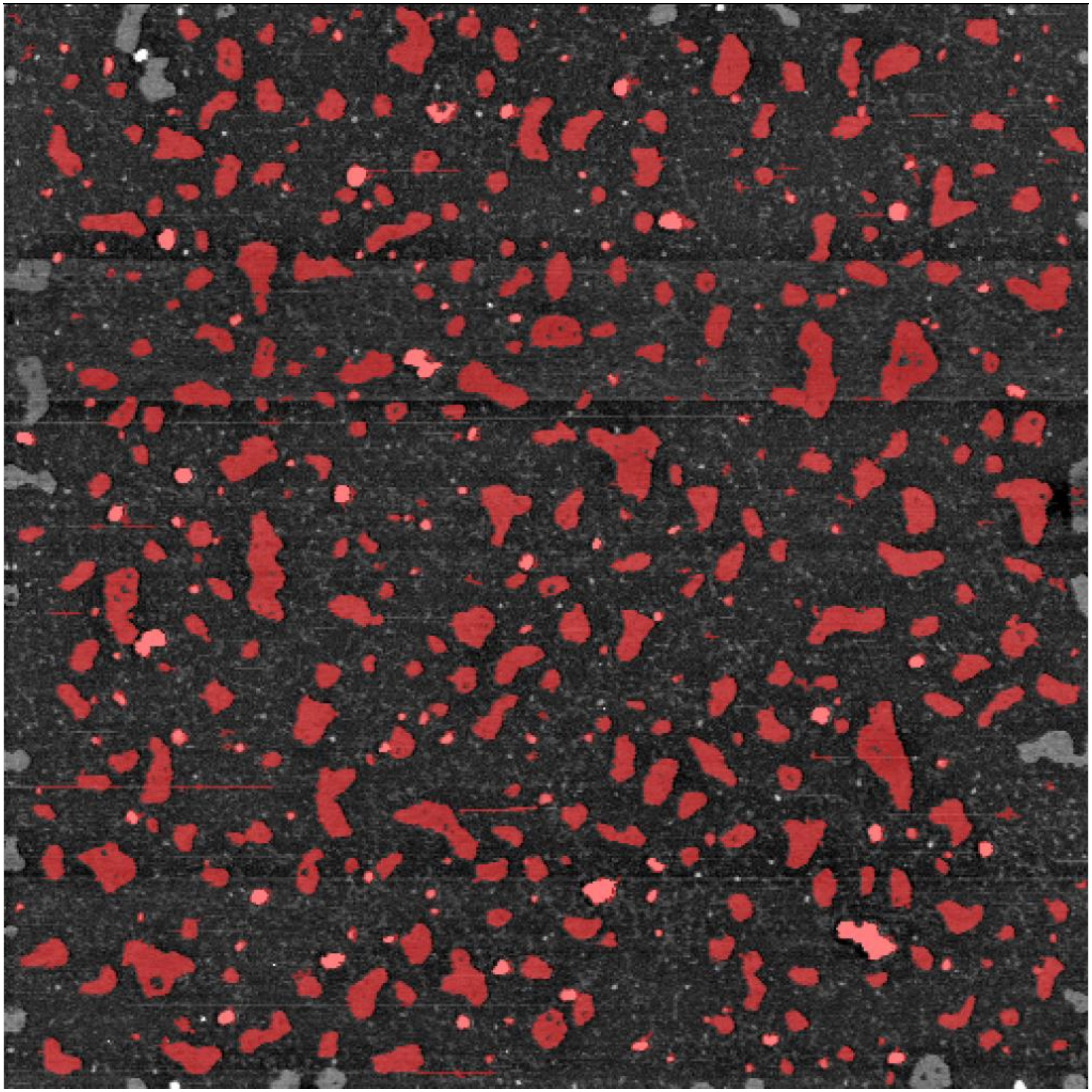

Figure S8. Grain Processed AFM images used for statistical analysis (Box size 2 μm)

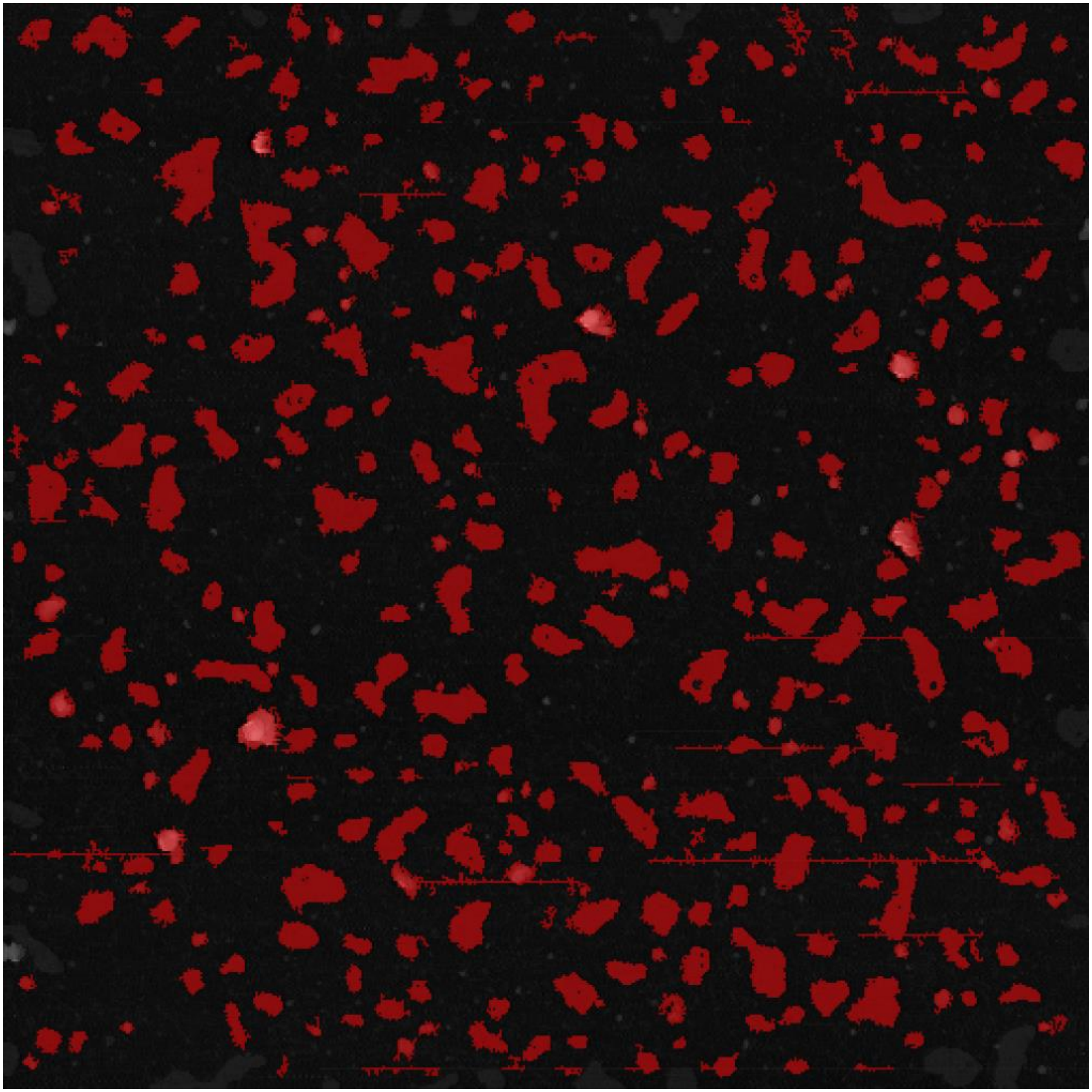

Figure S9. Grain Processed AFM images used for statistical analysis (Box size 2 µm)

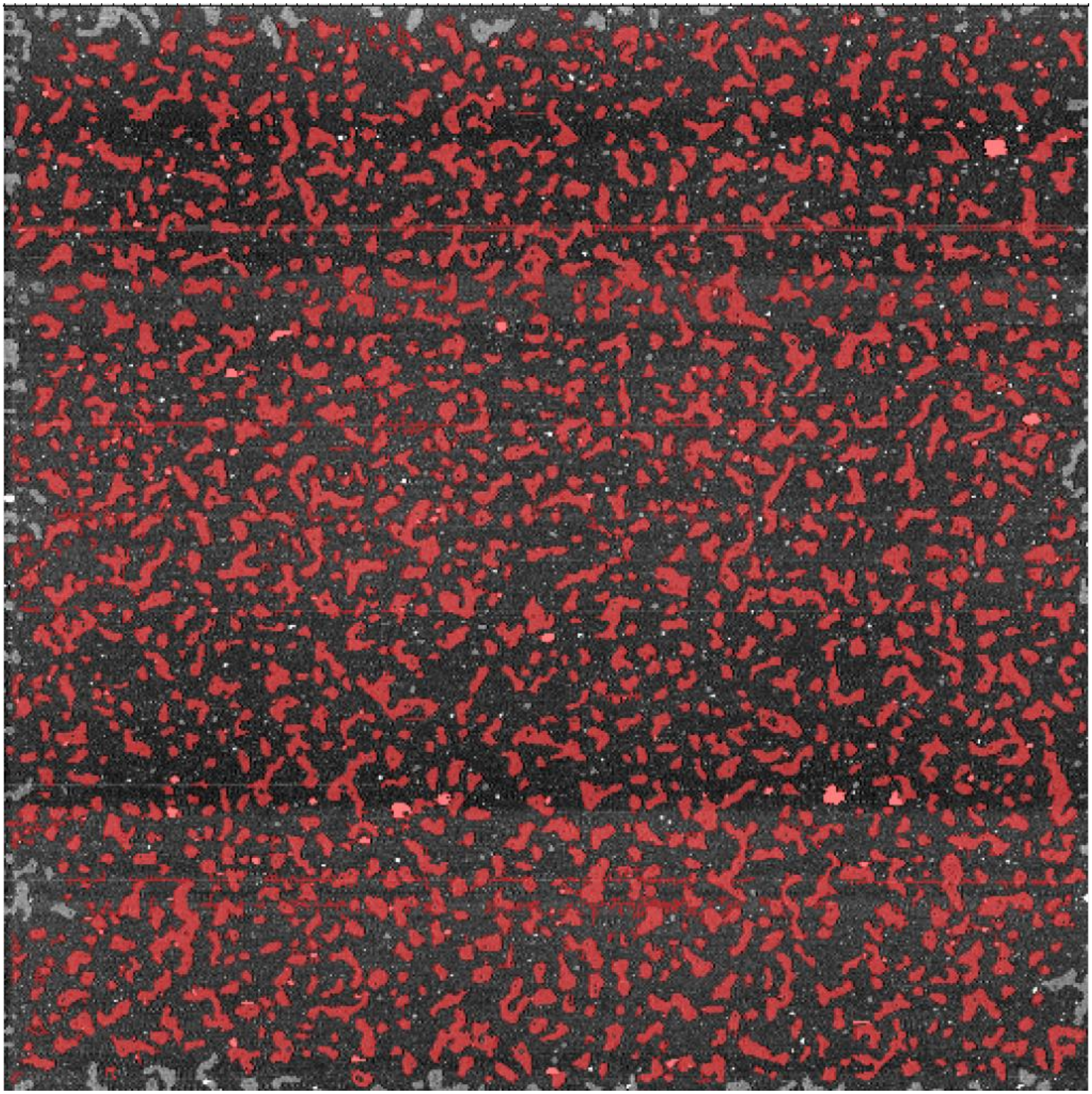

Figure S10. Grain Processed AFM images used for statistical analysis (Box size 5 µm)

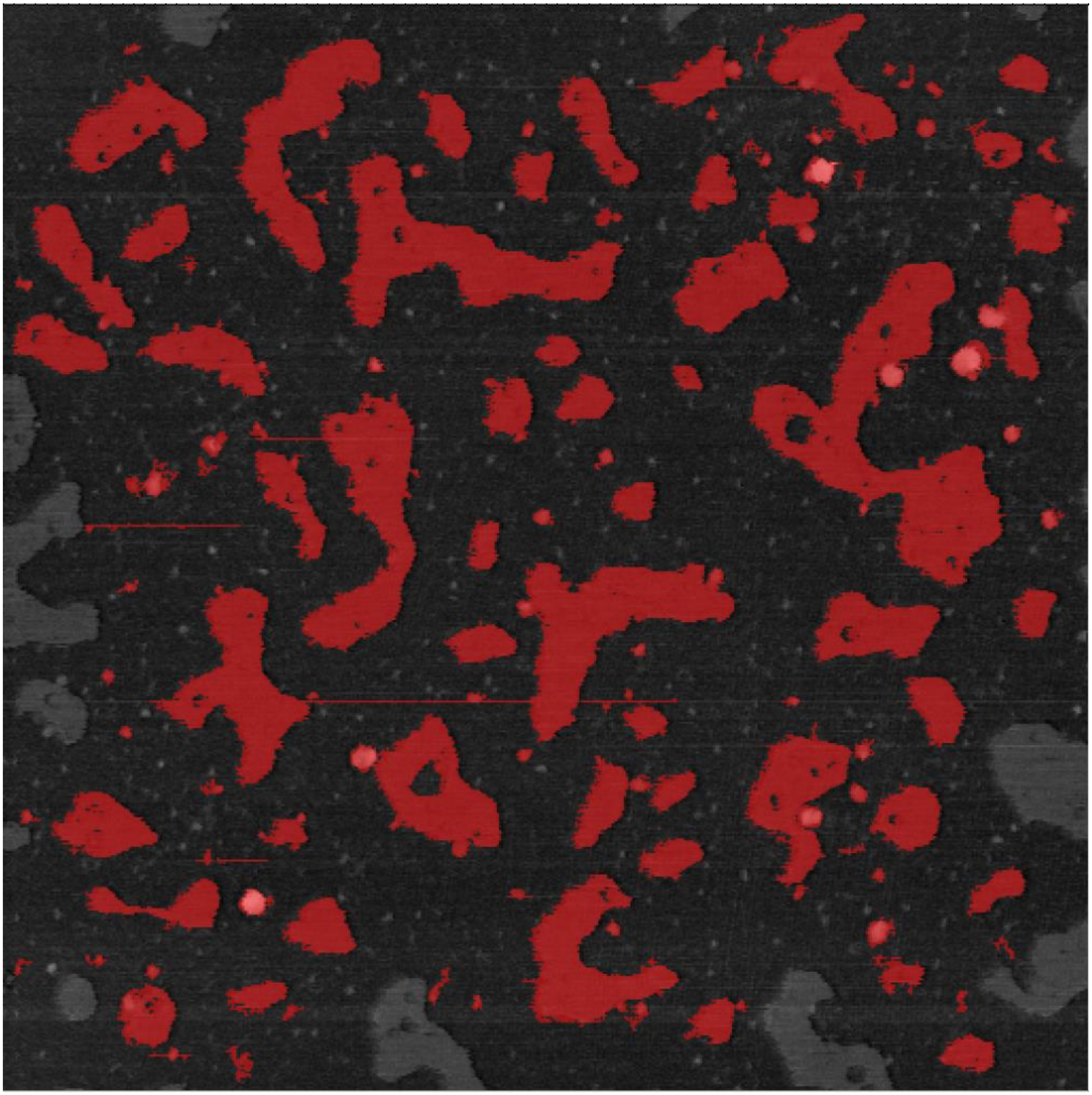

Figure S11. Grain Processed AFM image used for statistical analysis (Box size 1 µm)

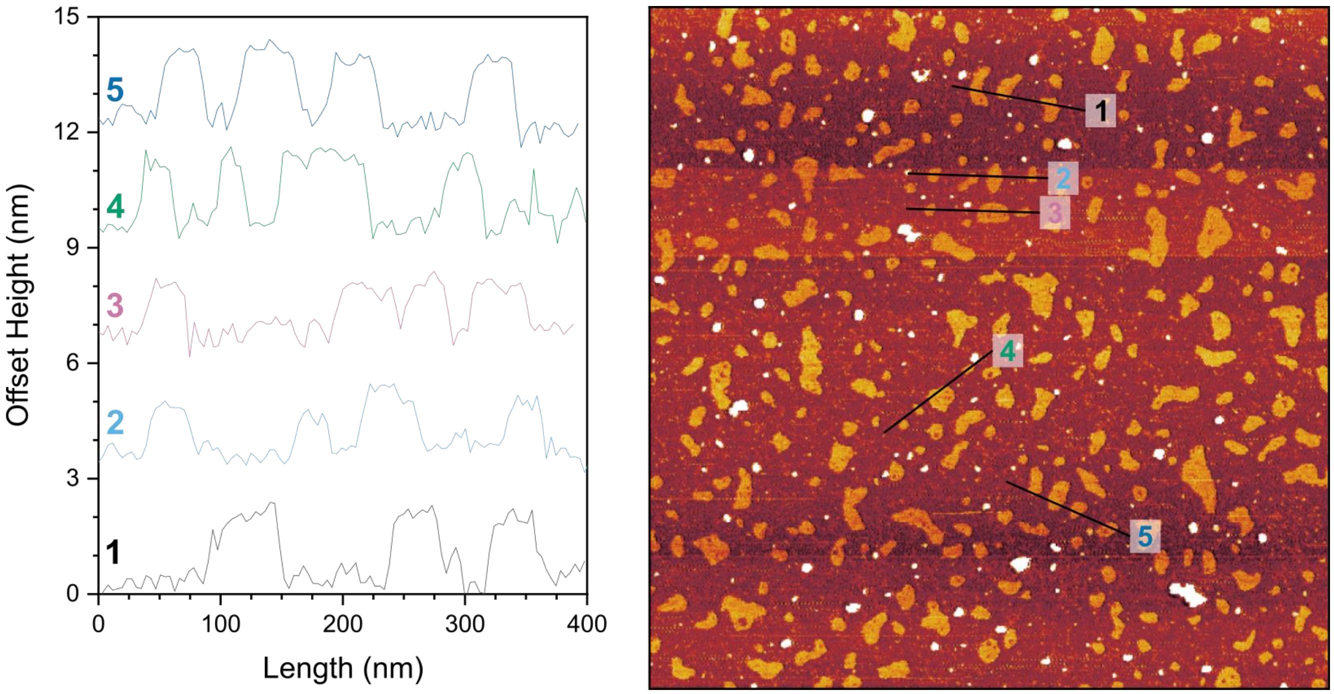

Figure S12. Line cuts of BPQD AFM micrograph, offset in 3 nm increments, with associated micrograph (Box size 2 μm, z-scale -1 to 4 nm)

Section S3. Data processing of the Liquid Phase Electron Microscopy data

Liquid phase EM is notoriously low signal to noise and very challenging to discern the signal from fluctuations in the noise from the low electron flux conditions. Over time an expect liquid phase TEM user can quickly discern the differences of specimen observations from variations in the noise of an image. However, for the purposes of a publications this is not the most compelling of data, we have therefore processed the data to assist the reader in identifying the quantum dots in the LPTEM measurements. All data was processed with SimpliPyTEM (https://github.com/gabriel-ing/Micrograph-analysis-scripts).

Topaz denoising is an algorithm[S1] used in CryoEM to suppress the signal from the amorphous ice layer from the images was applied to the LPTEM video (figure S15) The larger quantum dots can be more clearly been identified on the frames of the video.

For further clarity on the movement of the quantum dots in the liquid a sub-section of the video from figure S15 has been selected and a time series of the images shown in figure S16 more clearly illustrates the movement of the quantum dots in the liquid phase.

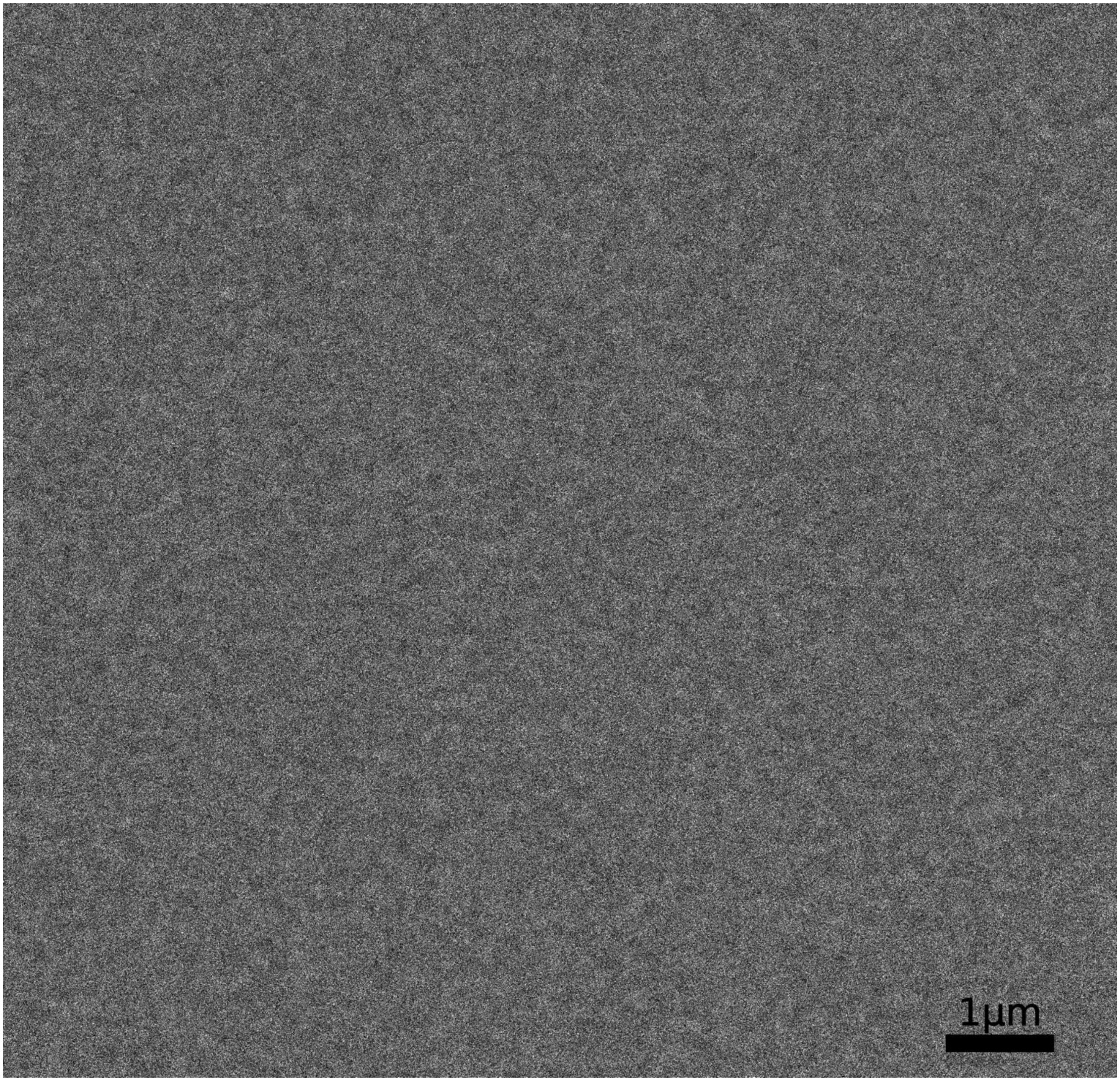

Figure S13. *Frame zero from the original data, see video S1 for original data with a gaussian filter*

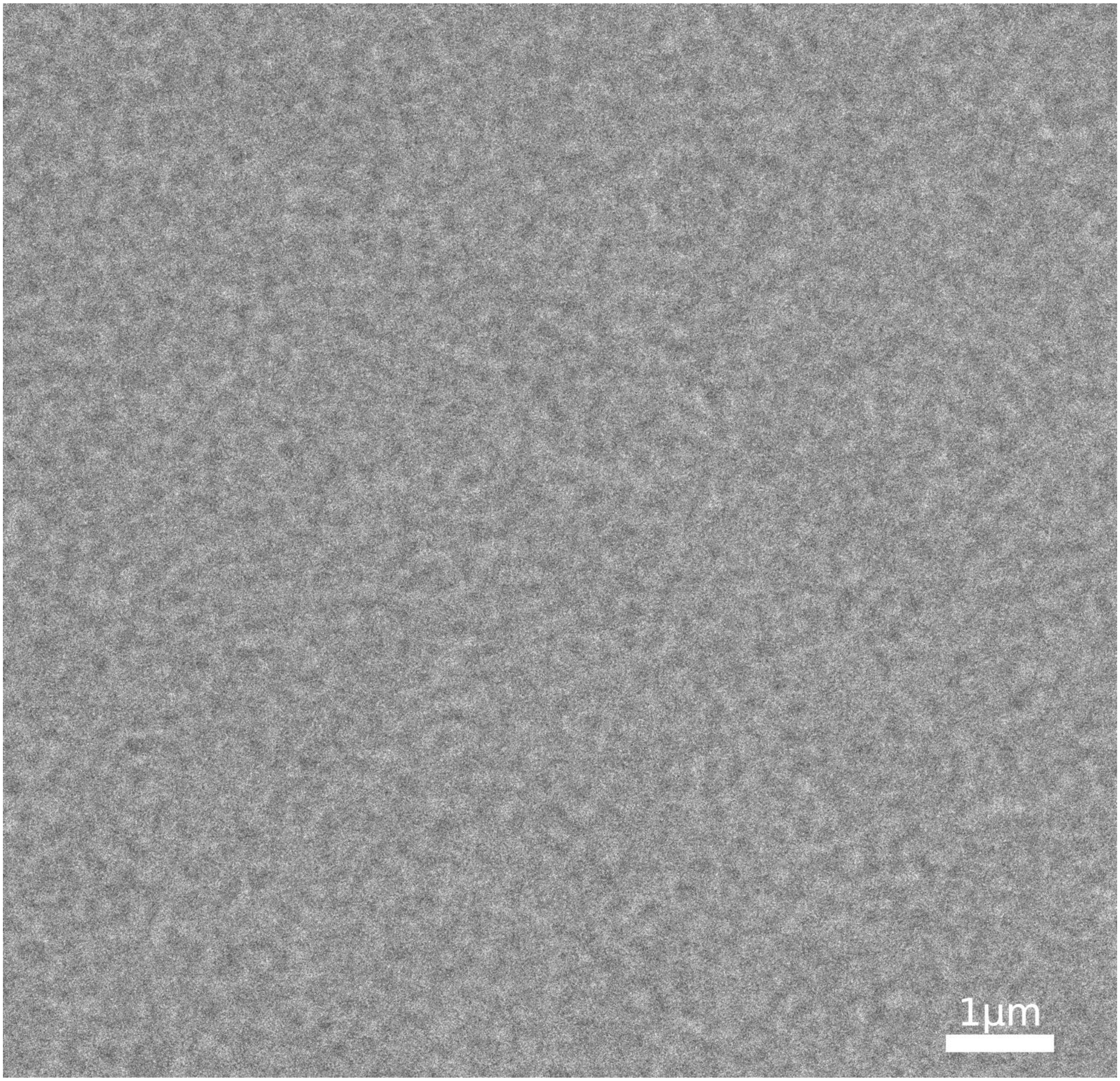

Figure S14: *A 5 frame averaging of the data was used to enhance features which relatively static during the data acquisition.* ***See video S2***

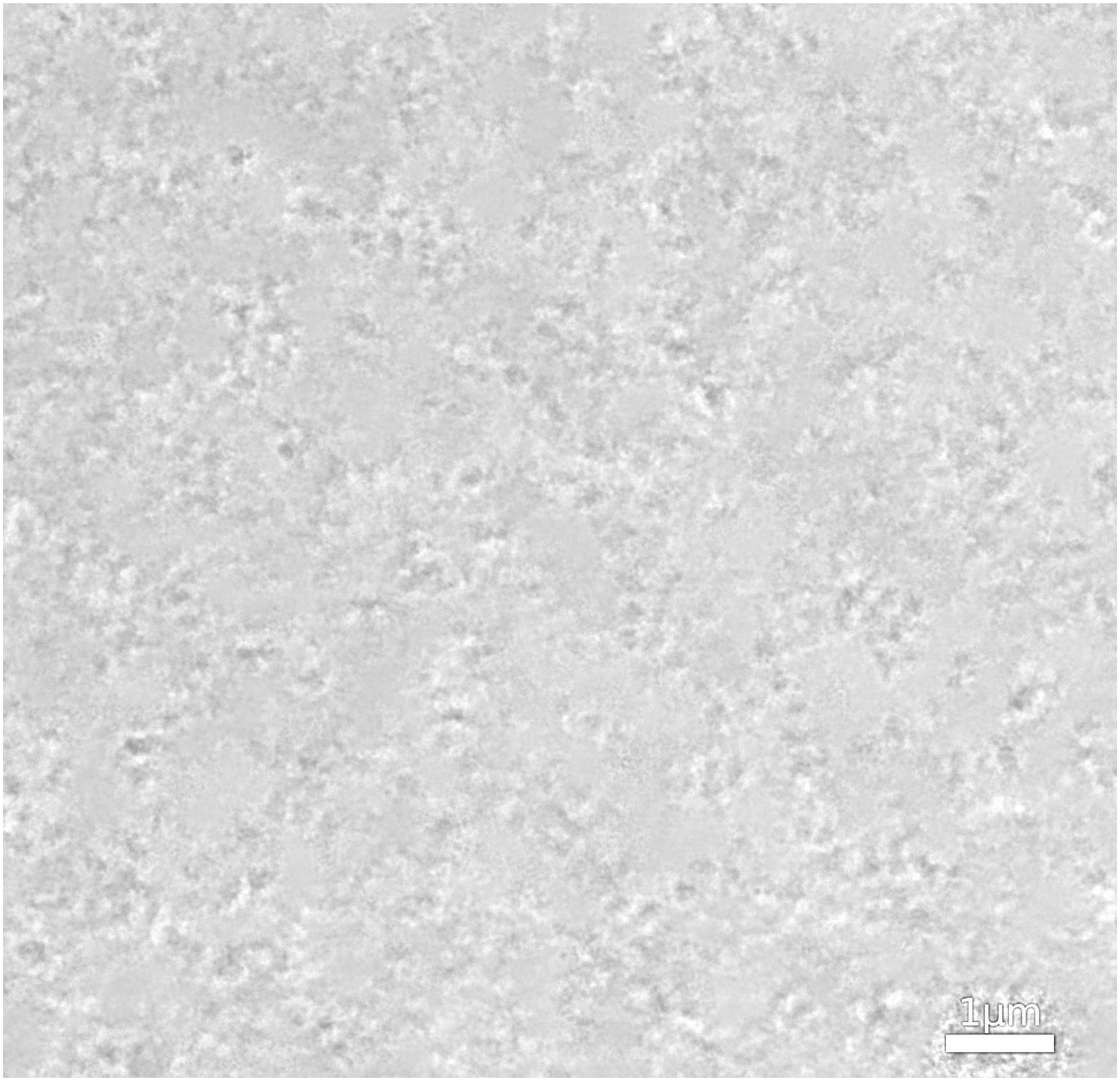

Figure S15. *Topaz demoing algorithm applied to the 5 frame average data.* ***See video S3***

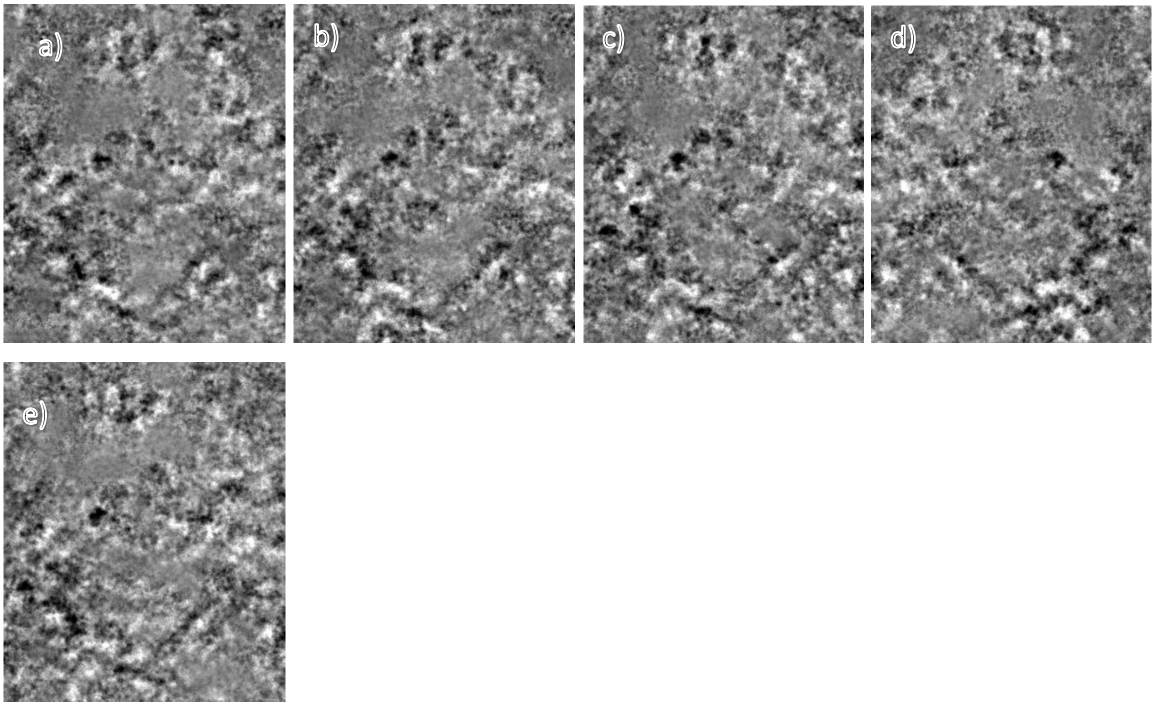

Figure S16: *A cropped region from the averaged and denoised frames of the videos to highlight the movement of the phosphorene quantum dots with time (a-e). See movie S4*

Section S4. A Practical Guide to Lithium Electride

We hope the simple nature of the BPQD synthesis reported herein will allow many researchers across disciplines to obtain and work with BPQDs. Many trained chemists will have experience in handling ammonia or comparable hazardous gases and their condensation, however, below we have provided a guide to some of the key hazards and considerations to aid less experienced scientists. If at all possible, please try to work alongside an experienced experimentalist acquainted with these processes.

Ammonia is a toxic and corrosive gas, and all work should be undertaken in a fume hood. For transfer of the anhydrous NH_3 gas, we use stainless steel Swagelok maintained at 10^{-7} mbar when not in use (N.B. ammonia will corrode non-dry steel tubing), but many common plastic tubings are in theory suitable. We explicitly note users should ***NOT*** use silicone-based or Viton tubing. Please check chemical compatibility charts on tubing before use. The gas system used should be fitted with an emergency release valve, vacuum line, and pressure gauge(s) to allow monitoring of pressures between 0.01 mbar to 3 bar. Use of a regulator to control ammonia pressure is recommended. A large empty flask (>200 mL) is recommended to be fitted to buffer fluctuations in pressure. Scientists should undertake work with a suitable respirator within reach in case of accidental release in addition to typical PPE.

To condense ammonia, a temperature of between -77 to -33 °C (196 – 306 K) is required which may be readily attained using a commercial chiller unit or dry ice cooling bath. Notably, dry ice with acetone/ethanol/isopropyl alcohol will not be suitable as it goes too cold (-78 °C) and is at risk of freezing the ammonia. Suitable common solvent/dry ice cold baths include acetonitrile (-46 °C), m-Xylene (-47 °C), n-octane (-56 °C), and chloroform (-61 °C), while safer alternatives include cyclohexanone (-46 °C) and diethyl carbitol (-52 °C).

For the condensation itself, firstly the system should be evacuated, then filled with an aliquot of ammonia, 1 atm is suitable and a small overpressure will encourage condensation ***only if the system is known to be safe at > 1 atm pressure***, and so long as the inert system is sealed and contained in a

fumehood (e.g. we operate our Swagelok system at ~3 bar, with a 5 bar release valve. These pressures are not suitable for a typical glass Schlenk line where the maximum internal pressure should be ~1 bar). We remind the reader that a suitable pressure relief valve and either a regulator or a gauge to measure overpressures is essential for the gas handling system. The pressure will fall as ammonia condenses in the cooled sample flask and eventually stabilise as the gas pressure approaches the vapour pressure of the ~-50 °C liquid ammonia (~0.4 bar). At this point, if insufficient ammonia has condensed to submerge the sample, another aliquot of ammonia gas should be added to increase the system pressure.

Upon condensation over Li metal, the ammonia will initially appear as a yellow metallic liquid – the so-called 'lithium bronze' which is the phase of high concentration Li in ammonia. As more ammonia is condensed, it will transform into a (non-metallic looking) dark blue solution of lithium electride. The solution may first be made then transferred into the Milled-P containing flask, but we typically simply condense the ammonia over both Li/Milled-P.

After the reaction is complete, the ammonia may be recovered into a lecture bottle (either containing NH_3, or under vacuum) to be reused for future reactions. To do so, first (i.e. before opening the bottle), the bottom half of the lecture bottle should be cooled with liquid nitrogen to freeze any NH_3 in the bottle. After, slightly open the bottle and observe the pressure, which should drop as gas is pulled into the bottle. If it rises, the gas inside has not sufficiently frozen and the bottle should be closed and cooled further. Once the pressure drops, the lecture bottle should continue to be cooled with liquid nitrogen with the bottle open. The reaction flask (containing BPQDs) should remain in the cooling bath throughout, allowing slow and careful evaporation from the low pressure concurrent with slow careful freezing in the lecture bottle. It may be unsafe to remove from the cooling bath, as it may lead to vigorous boiling of ammonia and a sharp increase in pressure. Once all liquid ammonia has been safely evaporated, allow the pressure to drop and stabilise – a trace residual atmosphere of trace NH_3 and H_2 (from slow Li degradation of NH_3) is unavoidable. Vacuum the entire system (lecture bottle and sample) and then close the lecture bottle. Only now remove the sample from the cooling bath and leave under vacuum to remove trace ammonia as it heats to room temperature.

Reference

[38] Bepler, T., Kelley, K., Noble, A.J., Berger, B. Topaz-Denoise: general deep denoising models for cryoEM and cryoET. Nat Commun 11, 5208 (2020). https://doi.org/10.1038/s41467-020-18952-1
